# Progressive hypothalamic neuroinflammation in ovariectomized mice parallels aging-related transcriptomic changes in the female human hypothalamus

**DOI:** 10.1172/jci.insight.207270

**Published:** 2026-07-23

**Authors:** Jordana C.B. Bloom, Encarnación Torres, Sidney A. Pereira, Liliana Arvizu-Sanchez, Audrey N. Fontes, Hadine Joffe, David C. Page, Victor M. Navarro

**Affiliations:** 1 Whitehead Institute for Biomedical Research, Cambridge, Massachusetts, USA.; 2 Division of Endocrinology, Mass General Brigham, Boston, Massachusetts, USA.; 3 Harvard Medical School, Boston, Massachusetts, USA.; 4 Department of Psychiatry, Beth Israel Deaconess Medical Center, Boston, Massachusetts, USA.; 5 Department of Biology, Massachusetts Institute of Technology, Cambridge, Massachusetts, USA.; 6 Howard Hughes Medical Institute, Cambridge, Massachusetts, USA.; 7 Harvard Graduate Program in Neuroscience, Boston, Massachusetts, USA.

**Keywords:** Endocrinology, Neuroscience, Reproductive biology, Neuroendocrine regulation

## Abstract

The hypothalamic changes that occur after the loss of ovarian estrogen remain poorly characterized. Here, we performed a comprehensive temporal characterization of the mouse hypothalamus after ovariectomy (OVX), combining physiological measurements with bulk RNA-seq of the posterior hypothalamus (PH) and preoptic area at 14 days and 4 months after OVX. Serum luteinizing hormone levels rose progressively and then declined, and core temperature peaked early and subsequently normalized, recapitulating the endocrine and thermoregulatory dynamics of reproductive aging in humans. Transcriptomic analysis revealed time-dependent activation of inflammatory pathways, glial markers, and KNDy neuron-related gene networks, with the most pronounced changes emerging at 4 months after OVX, particularly in the PH. Immunofluorescence confirmed increased neurokinin B release, declining KNDy neuronal activity, and heightened astrocytic reactivity in the arcuate nucleus after prolonged estrogen withdrawal. To contextualize these findings, we analyzed publicly available human hypothalamic RNA-seq data across chronological age. Age-related transcriptomic patterns, including progressive inflammatory signaling, glial activation, and altered KNDy gene expression, showed significant correlation with the OVX mouse model, particularly at the pathway level. These findings establish a temporal framework for hypothalamic molecular changes after estrogen withdrawal, identify conserved neuroinflammatory signatures across species, and provide a preclinical platform for testing interventions targeting menopause-associated hypothalamic dysfunction.

## Abstract 2

Estrogen loss triggers progressive brain inflammation in the hypothalamus, with mouse changes mirroring those in aging women, offering new insight into menopause and its symptoms.

## Introduction

Menopause represents a significant life transition for women, characterized by physiological changes extending beyond reproductive function (1). Understanding the neurobiological basis of these changes is crucial for advancing women’s health and developing targeted interventions for menopausal symptoms (2). Although much attention has been focused on ovarian function, research focused on characterizing the changes that happen at the level of the brain, particularly the hypothalamus, has lagged. The hypothalamus contains neuroendocrine centers that both control and respond to estrogens (3, 4), and hypothalamic changes are implicated in disrupted thermoregulation, sleep patterns, and increased mood disorders during menopause (5–12).

Hypothalamic KNDy neurons (expressing kisspeptin, neurokinin B [NKB], and dynorphin A) have emerged as central players in vasomotor symptoms (VMS), including hot flashes and night sweats, which are among the most common and disruptive menopausal symptoms (13, 14). The identification of KNDy neurons as VMS triggers has opened new avenues for understanding and treating these symptoms (13–15), ultimately leading to the FDA approval of NK3R antagonists for VMS (16). However, our knowledge of broader hypothalamic changes during and after estrogen withdrawal remains incomplete, compounded by limitations in current animal models (17).

A critical gap in the field has been the absence of a well-characterized temporal model of hypothalamic changes after estrogen loss. Most ovariectomy (OVX) studies in rodents examine a single short-term time point, typically 1–3 weeks after surgery, which captures the acute response to estrogen withdrawal but fails to model the progressive changes that develop over months to years in women. This is particularly relevant because VMS and other menopausal symptoms show distinct temporal trajectories. VMS typically peak during the perimenopausal period and gradually resolve years after menopause, while inflammatory and neurodegenerative processes may continue to progress (18). Whether these clinically distinct phases correspond to discrete molecular programs in the hypothalamus has not been systematically investigated.

Here, we address this gap by performing a comprehensive temporal analysis of the mouse hypothalamus at multiple time points after OVX, integrating endocrine measurements, core body temperature, bulk RNA-seq of 2 hypothalamic subregions, and protein-level validation. We then leveraged publicly available human hypothalamic transcriptomic data from the Genotype-Tissue Expression (GTEx) consortium to assess whether the mouse molecular signatures parallel age-related changes observed in women across the adult life span, while acknowledging the inherent limitations of the human observational dataset. By combining rigorous experimental data with complementary human observations, this study provides both mechanistic insight and a preclinical platform for developing interventions targeting hypothalamic dysfunction after estrogen withdrawal.

## Results

### Progressive endocrine and thermoregulatory changes after OVX in mice.

**Figure 1:**
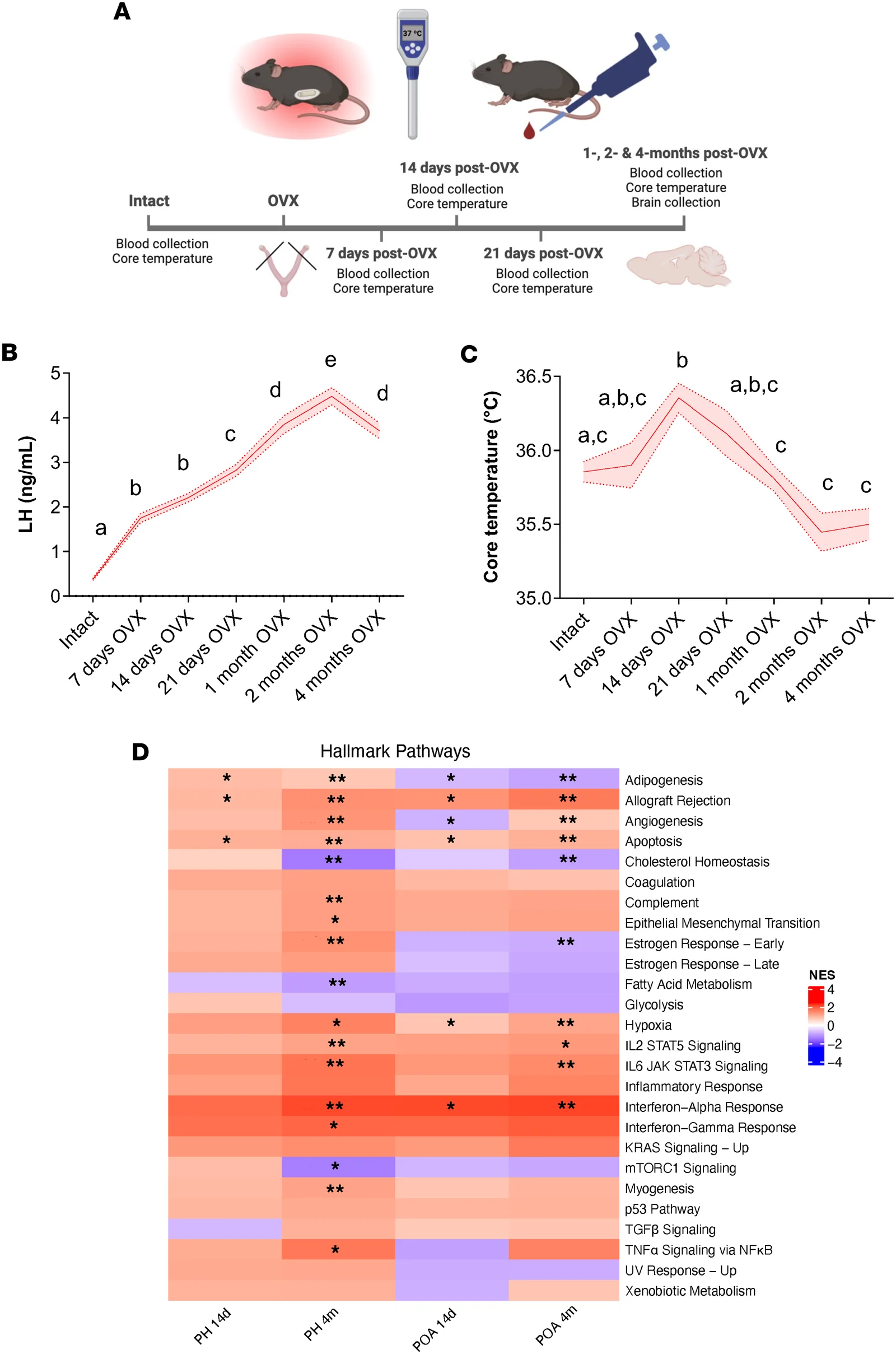
Progressive physiological and transcriptomic changes in the mouse hypothalamus after OVX. (**A**) Schematic representation of the experimental design. (**B**) LH concentration in serum from whole blood at 7, 14, and 21 days and 1, 2, and 4 months after OVX in WT female mice (*n* = 20/group). (**C**) Core temperature (°C) measurements at 7, 14, and 21 days and 1, 2, and 4 months after OVX in WT female mice (*n* = 20/group). (**B** and **C**) Data shown as mean ± SEM. Groups with different letters are significantly different; *P* < 0.05 by 1-way ANOVA and Tukey’s post hoc test. (**D**) Heatmap of normalized enrichment scores for Hallmark gene sets in the PH and POA; significant enrichments indicated by asterisks (*adjusted *P* < 0.05; **adjusted *P* < 0.01; ***adjusted *P* < 0.001).

To model the time-dependent effects of estrogen withdrawal on the hypothalamus, we assessed a series of time points after OVX in WT female mice (Figure 1A). Serum luteinizing hormone (LH) concentrations increased progressively, with levels peaking at 2 months after OVX before declining at 4 months after OVX (Figure 1B). This biphasic LH pattern, initial rise followed by decline, parallels the endocrine trajectory observed during human reproductive aging, in which gonadotropin levels increase during perimenopause and subsequently decline in the late postmenopause stage (19). To characterize changes in thermoregulation, core temperature was measured across time points, revealing a peak at 14 days after OVX that progressively normalized by 4 months (Figure 1C). Together, these physiological data indicate that the 14-day post-OVX model captures the acute neuroendocrine response to estrogen withdrawal, characterized by elevated gonadotropins and disrupted thermoregulation, and the 4-month post-OVX model reflects a more chronic, adapted state with declining gonadotropins and normalized temperature.

### Time-dependent hypothalamic transcriptomic changes after OVX.

To determine whether these physiological changes are accompanied by corresponding molecular alterations in the hypothalamus, we performed bulk RNA-seq of 3 models: intact, a 14-day (short-term) post-OVX model, and a 4-month (long-term) post-OVX model. We selected 2 regions of the hypothalamus based on the localization of neurons controlling temperature regulation (preoptic area, POA) and the localization of neurons controlling metabolic and reproductive centers (posterior hypothalamus, PH). Cell-type deconvolution analyses of the bulk RNA-seq data revealed the presence of previously reported hypothalamic cell types (20) across all samples (Supplemental Figure 1; supplemental material available online with this article; https://doi.org/10.1172/jci.insight.207270DS1).

Global gene set enrichment analysis (GSEA) (21) revealed marked enrichment in inflammatory pathways, with the greatest changes observed at 4 months after OVX in the PH and POA (Figure 1D and Supplemental Table 1). TNF-α signaling via NF-κB, IFN responses, IL6/JAK/STAT3 signaling, and complement pathways were among the most significantly enriched. STRING analysis (22) of TNF-α hallmark pathway leading-edge genes highlighted genes related to thermoregulation (23), including positive regulation of fever generation and prostaglandin biosynthesis (24) (Supplemental Figure 2). Notably, estrogen response pathways showed progressive depletion with time after OVX, particularly in the POA, consistent with the loss of ovarian estrogen signaling. These data demonstrate that the hypothalamic inflammatory response to estrogen withdrawal is not immediate but rather develops progressively, with the most robust transcriptomic changes emerging months after OVX.

### Differential gene expression reveals distinct short-term and long-term OVX signatures.

**Figure 2:**
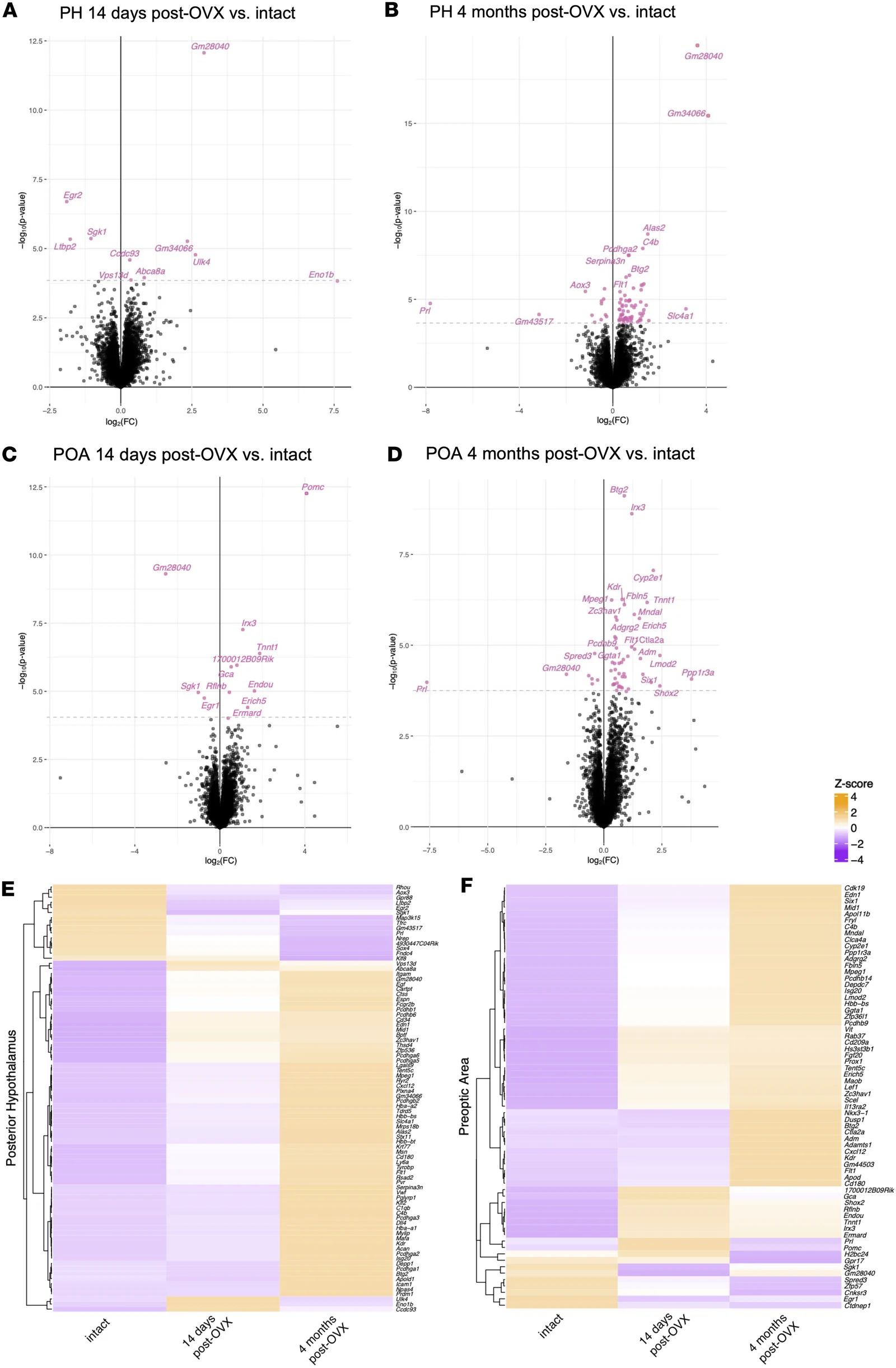
Differential gene expression in the post-OVX mouse hypothalamus. (**A**–**D**) Volcano plots highlighting significant DEGs (FDR < 0.05, labeled in pink) between (**A**) PH 14 days after OVX versus intact (13 protein-coding DEGs), (**B**) PH 4 months after OVX versus intact (82 protein-coding DEGs), (**C**) POA 14 days after OVX versus intact (14 protein-coding DEGs), (**D**) POA 4 months after OVX versus intact (70 protein-coding DEGs). (**E**) Expression of PH DEGs across groups. (**F**) Expression of POA DEGs across groups.

We next conducted differential gene expression (25) analyses between the 3 models: the intact model, short-term post-OVX model, and long-term post-OVX model in both the PH and POA. Volcano plots highlight significantly differentially expressed genes (DEGs) (FDR < 0.05) between conditions and regions (Figure 2, A–D; Supplemental Figure 3, A and B; and Supplemental Table 2). Notably, the number of DEGs increased substantially at 4 months relative to 14 days after OVX. Heatmaps depicting how the identified genes are expressed across conditions in the PH (Figure 2E) and POA (Figure 2F and Supplemental Table 3) revealed distinct temporal profiles: 27 protein-coding genes were already altered at 14 days after OVX, and 143 showed changes only at 4 months; 9 showed progressive changes across both time points.

Among the DEGs, *Gm28040*, which shares overlapping coding sequence with *Kiss1*, showed the largest expression change, increased at 14 days and 4 months after OVX in the PH and decreased in both groups in the POA, resembling the known region-specific regulation of *Kiss1* in both hypothalamic areas (26). This suggests that in the mouse, *Gm28040* encodes a kisspeptin isoform (Figure 2, A–D, and Supplemental Table 2).

We then examined the expression of previously identified marker genes for inflammation (Supplemental Figure 4A) and reproduction (Supplemental Figure 4B) in both the PH and the POA. Although individual KNDy and inflammation-related genes did not reach statistical significance after adjustment for multiple hypothesis testing, the expression of inflammation and astrocytic markers showed increasing trends from intact to 4 months after OVX, with notable increases in *Vim*, *Iba1*, and *Tspo* in the PH and in the prostaglandin pathway (*Ptgs2*) in the POA. The KNDy genes (*Kiss1*, *Tac2*, and *Pdyn*), involved in reproductive and thermoregulatory (VMS) control, showed a trend toward higher expression in the PH (where the arcuate nucleus is located) in proportion to time after OVX, consistent with progressive loss of estrogen-negative feedback on these sex steroid–regulated genes (27).

### Protein-level validation of KNDy neuron changes and neuroinflammation.

**Figure 3:**
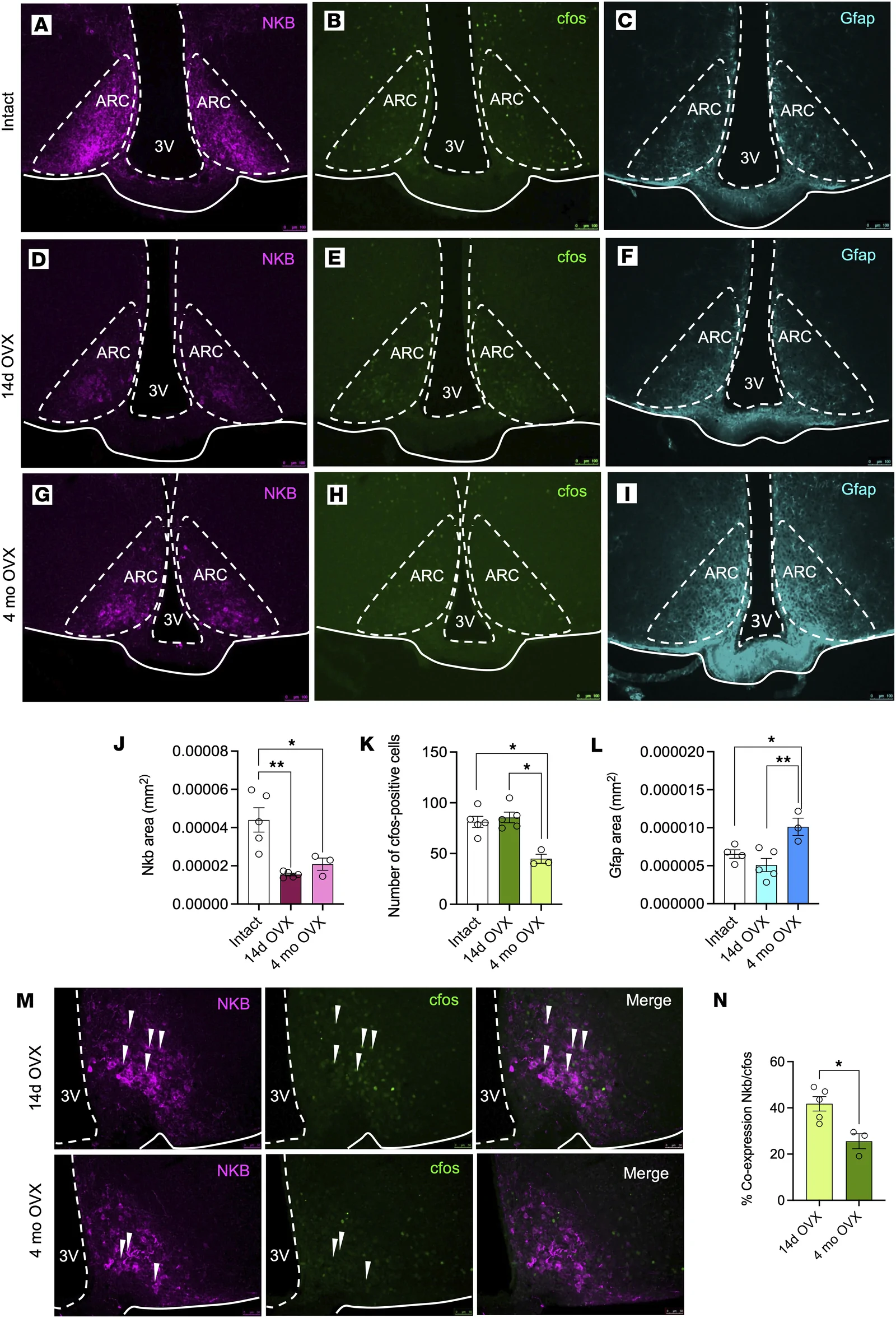
Differential NKB distribution, neuronal activity, and astrocyte reactivity in the arcuate nucleus after OVX. Representative immunofluorescence staining of NKB (**A**, **D**, and **G**), cFos (**B**, **E**, and **H**), and GFAP (**C**, **F**, and **I**) in the arcuate nucleus (ARC) from intact (**A**–**C**), 14-day post-OVX (**D**–**F**), and 4-month post-OVX mice (**G**–**I**, *n* = 3–5/group). (**J**) Quantification of NKB area (mm^2^) in the ARC from intact, 14-day post-OVX, and 4-month post-OVX mice. (**K**) Quantification of cFos-positive cells in the ARC from intact, 14-day post-OVX, and 4-month post-OVX mice. (**L**) Quantification of GFAP area (mm^2^) in the ARC from intact, 14-day post-OVX, and 4-month post-OVX mice. (**M**) Representative images of NKB and cFos colocalization in the ARC from 14-day and 4-month post-OVX mice (*n* = 3–5/group). (**N**) Percentage of activated (cFos^+^) cells coexpressing NKB. Data from **J**–**L** and **N** shown as mean ± SEM. Statistical significance determined by Mann-Whitney *U* test (intact vs. OVX). *P < 0.05, **P < 0.01.

To validate key transcriptomic findings at the protein level, we performed immunofluorescence staining for NKB, the early activation marker c-Fos, and the astrocyte marker glial fibrillary acidic protein (GFAP) in the arcuate nucleus (Figure 3). NKB immunoreactivity was significantly reduced in both 14-day and 4-month post-OVX mice compared with intact controls (Figure 3, A, D, G, and J). This decrease in NKB peptide stores, in the context of increased *Tac2* mRNA expression (Supplemental Figure 4B), is consistent with enhanced neuropeptide release and depletion rather than decreased synthesis, a hallmark of hyperactivated neuropeptide systems.

c-Fos expression in the arcuate nucleus remained elevated at 14 days after OVX at a similar level to control gonad intact mice and declined significantly by 4 months (Figure 3, B, E, H, and K). To determine whether this decline in activity occurred specifically in KNDy neurons, we quantified the proportion of activated (c-Fos^+^) arcuate cells that coexpressed NKB, which revealed an approximately 50% decrease in KNDy-activated cells at 4 months compared with 14 days after OVX (Figure 3, M and N). This reduction in KNDy neuron activity at the long-term post-OVX time point aligns with the normalization of core body temperature observed at this time point (Figure 1C) and provides a cellular basis for the temporal dynamics of vasomotor-like responses.

GFAP immunoreactivity increased progressively with time after OVX, with significant elevations at both 14 days and 4 months compared with intact animals (Figure 3, C, F, I, and L). This increase in astrocytic reactivity corroborates the transcriptomic evidence for progressive neuroinflammation and glial activation in the hypothalamus after estrogen withdrawal.

### Age-related transcriptomic changes in the human female hypothalamus.

Having established the temporal dynamics of hypothalamic changes after experimental estrogen withdrawal in mice, we next sought to determine whether analogous molecular signatures could be identified in the human hypothalamus. We leveraged publicly available bulk RNA-seq data from the GTEx project (28) to examine hypothalamic gene expression changes across the adult female life span. It is important to note that this human dataset, although it is the best currently available resource, has an inherent limitation since samples derive from postmortem tissue of 31 female donors (ages 28–65) whose menopausal status and hormone therapy history are unavailable. We therefore present these findings as age-related transcriptomic patterns in the human hypothalamus, with the caveat that associations with menopausal status, although biologically plausible given the known timing of menopause, cannot be definitively established from this dataset alone.

Female donors with hypothalamic tissue were included if they met GTEx donor eligibility criteria and their sequenced tissue had an RNA integrity number of 6 or greater (Supplemental Table 4). The 31 samples span the adult female age range (Supplemental Figure 5, A and B). Surrogate variable analysis (SVA) (29) was performed on the RNA expression matrix prior to gene expression analyses to control for latent confounding sources of variation in the data, such as postmortem interval, donor cause of death, and hormone therapy status. Cell-type deconvolution of the bulk RNA-seq data revealed the presence of previously reported hypothalamic cell types (20) (Supplemental Figure 6A and Supplemental Table 5) and the presence of all 8 major transcription factors (*FEZF1*, *FOXB1*, *LHX6*, *MEIS2*, *OTP*, *SIM1*, *SIX3*, *TBX3*) identified in the human hypothalamus from the HYPOMAP single-nucleus RNA-seq reference atlas (30) (Supplemental Figure 6B), indicating that there are no major differences in cell-type composition between samples in the cohort.

**Figure 4:**
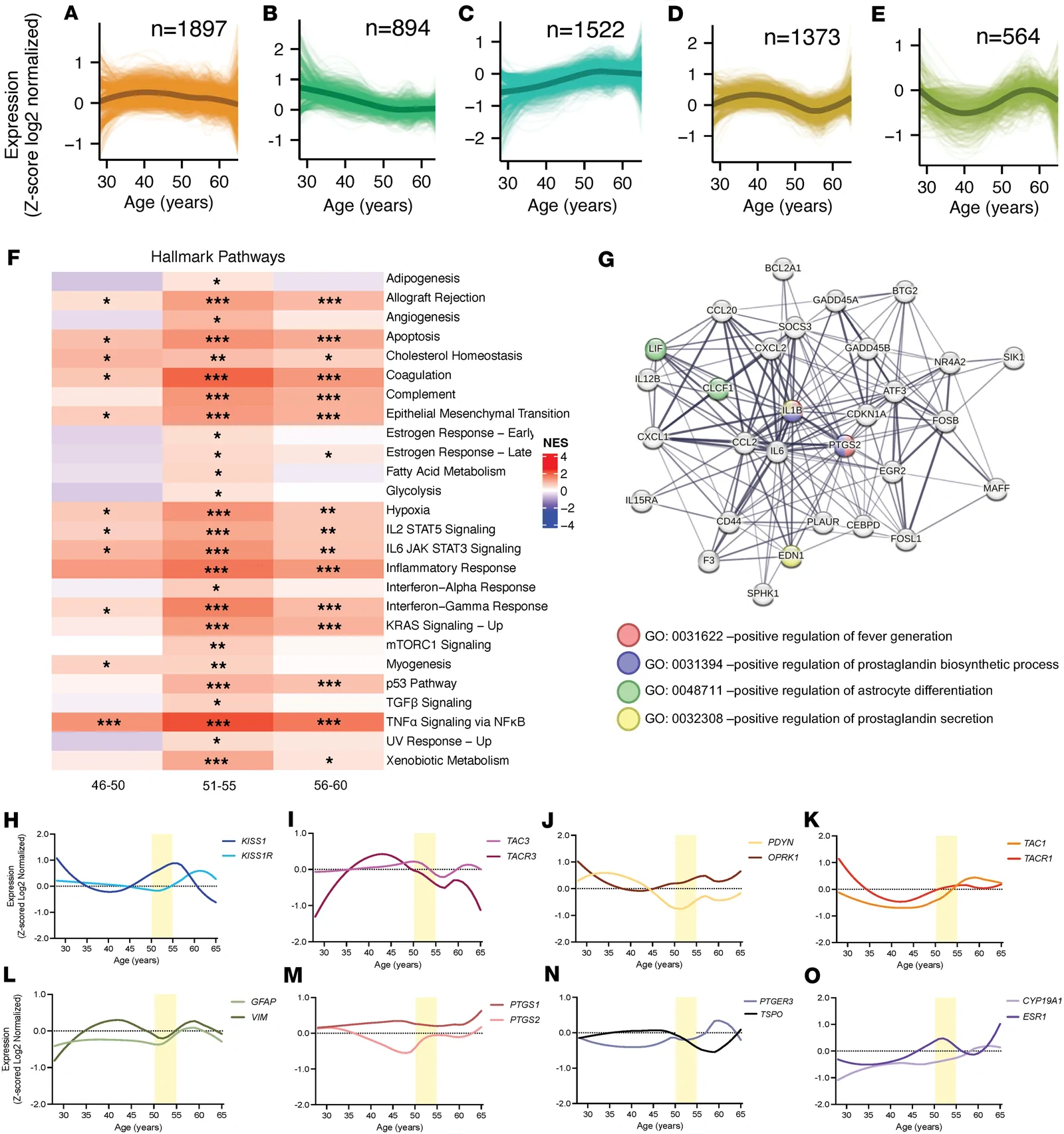
Gene expression trajectories and pathway analysis in the human female hypothalamus across chronological age. (**A**–**E**) Sets of genes grouped by hierarchical clustering and displayed using LOESS trajectories exhibit distinct patterns: (**A**) constant expression across age, (**B**) decreased expression across age, (**C**) increased expression across age, (**D**) decreased expression during ages 51–60, (**E**) increased expression during ages 51–60. (**F**) Heatmap of normalized enrichment scores for Hallmark gene sets in which there was a statistically significant pathway enrichment in at least 1 of the 3 age-group comparisons (46–50, 51–55, and 56–60 vs. <45); significant enrichments indicated by asterisks (*adjusted *P* <0.05; **adjusted *P* < 0.01; ***adjusted *P* < 0.001). (**G**) STRING analysis of TNF-α Hallmark pathway leading-edge genes between the 51–55 and younger than 45 age groups. (**H**–**O**) LOESS trajectories of KNDy genes and their receptors, thermoregulatory genes, and astrocyte- and microglia-marker genes across age in human hypothalamus tissue: (**H**) *KISS1* and *KISS1R*, (**I**) *TAC3* and *TACR3*, (**J**) *PDYN* and *OPRK1*, (**K**) *TAC1* and *TACR1*, (**L**) *GFAP* and *VIM*, (**M**) *PTGS1* and *PTGS2*, (**N**) *PTGER3* and *TSPO*, and (**O**) *CYP19A1* and *ESR1*.

We assessed patterns of gene expression changes across ages using locally estimated scatterplot smoothing (LOESS). LOESS expression profiles were grouped by hierarchical clustering, revealing distinct patterns, including genes with nearly constant (Figure 4A), decreased (Figure 4B), and increased (Figure 4C) expression across age. Of particular interest was the identification of gene clusters with specific decreases (Figure 4D) or increases (Figure 4E) coinciding with the age range during which menopause typically occurs (~45–55 years) (Supplemental Table 6), though we emphasize that without direct menopausal status data, we cannot attribute these changes solely to menopause rather than to other age-related processes.

Next, we divided the cohort into age bins informed by epidemiological data from the Study of Women’s Health Across the Nation (SWAN), where the median age at final menstrual period is 51–52 years (31) (Supplemental Figure 5B): younger than 45 years (*n* = 4), 46–50 years (*n* = 4), 51–55 years (*n* = 7), 56–60 years (*n* = 7), and 61–65 years (*n* = 9). We acknowledge that individual variation in menopausal timing means these bins represent chronological age ranges that statistically correspond to typical reproductive stages in population studies, such as SWAN, but we cannot assign clinical menopausal staging to individual donors. GSEA (21) between each age group and the younger than 45 group revealed the highest and most significant enrichments in inflammatory pathway signaling in the 51–55 age group (Figure 4F and Supplemental Table 7). These included TNF-α signaling via NF-κB, IFN responses, IL2/STAT5 and IL6/JAK/STAT3 signaling, and complement pathways, strikingly similar to the pathways enriched in the long-term OVX mouse model (Figure 1D). The 46–50 age group showed partial overlap with the 51–55 age group, albeit with lower normalized enrichment scores. In this perimenopausal-aged group, the most significantly enriched pathways relative to the younger than 45 group were the inflammatory response and TNF-α signaling via NF-κB pathways (both adjusted *P* < 0.001), with additional significant enrichment (adjusted *P* < 0.05) of IL2/STAT5 and IL6/JAK/STAT3 signaling, the IFN-γ response, hypoxia, and allograft rejection, indicating that the inflammatory transcriptional program is already emerging during this transition window. STRING analysis (22) of TNF-α hallmark pathway leading-edge genes between the 51–55 and younger than 45 age groups highlighted genes involved in thermoregulation (23, 24), including fever responses (Figure 4G).

We examined the LOESS trajectories of key ligand-receptor systems related to KNDy signaling, thermoregulation, and glial activation in the human hypothalamus across age groups (Figure 4, H–O). *KISS1* and *ESR1* showed marked increases during the typical menopausal years, consistent with loss of estrogen-negative feedback (32, 33), followed by a decline approximately 10 years after their peak. *TACR3* expression increased in the years leading up to age 50 and then progressively declined. The astrocyte activation markers *GFAP* and *VIM* increased during the 50–55 age range, and the microglial marker *TSPO* increased in later years, as previously reported (34). Changes in prostaglandin synthases (*PTGS1/2*) and receptors (*PTGER3*) were also observed, with *PTGER3* showing a notable increase followed by a sharp decrease around age 60. Based on a recent publication examining *PGR*, *GPER1*, and *ESR2* as potential menopause biomarkers from postmortem tissue (35), we also assessed the expression trajectory of these genes (Supplemental Figure 7). Although not significantly altered in the categorical analysis performed by Tickerhoof et al. (35), we observed that *GPER1* and *ESR2* decreased and *PGR* exhibited a notable inflection during the 50–55 age range.

### Identification of age-associated DEGs in the human hypothalamus.

**Figure 5:**
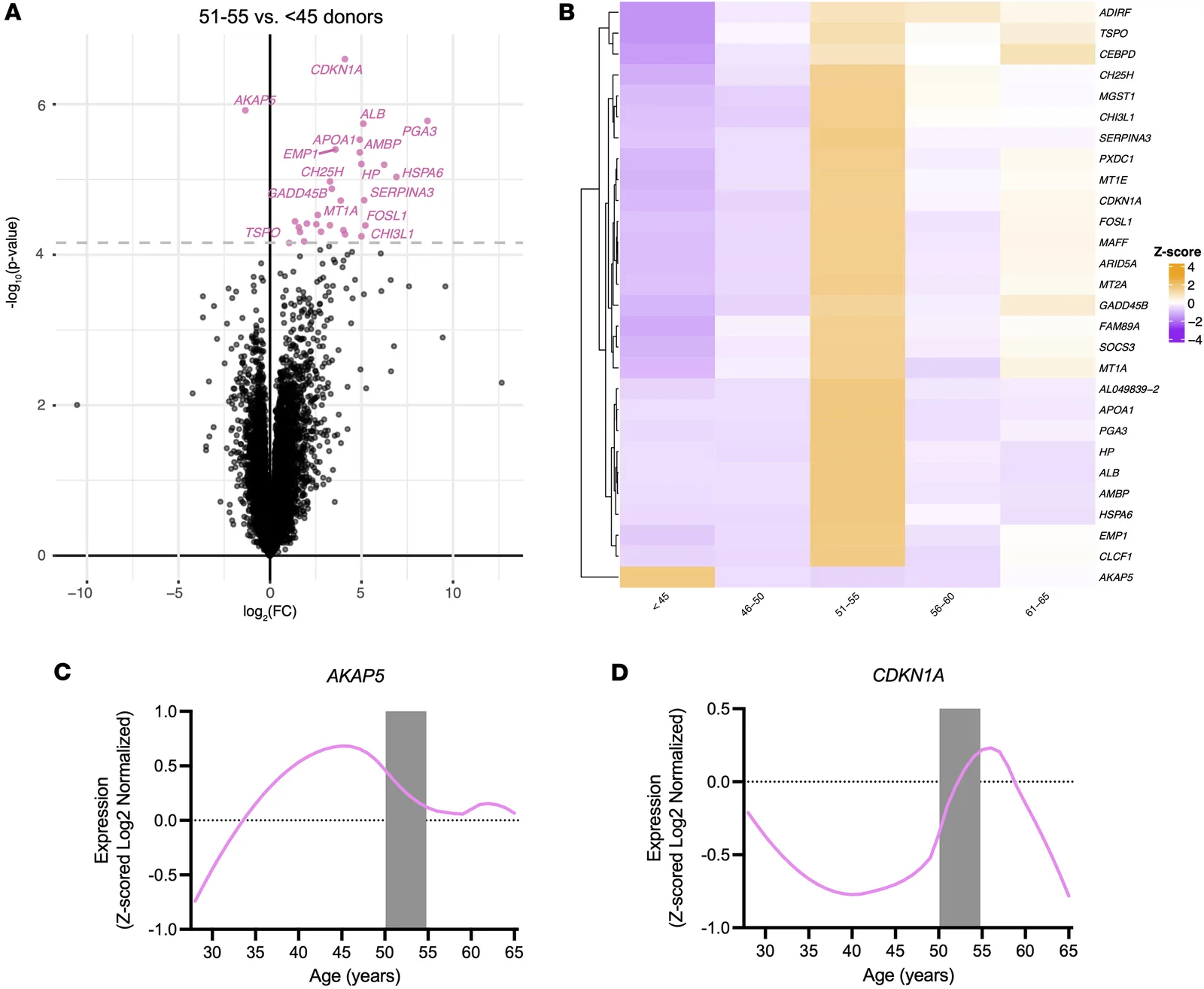
Age-associated DEGs in the human hypothalamus. (**A**) Volcano plot highlighting significant DEGs between the 51–55 and younger than 45 age groups. Genes with FDR less than 0.05 are highlighted in pink. (**B**) Expression of genes with FDR less than 0.05 across age groups. (**C**) LOESS trajectory of *AKAP5* across age. (**D**) LOESS trajectory of *CDKN1A* across age.

We performed differential gene expression analysis between the 51–55 and younger than 45 age groups (Figure 5A and Supplemental Table 8). Twenty-eight protein-coding genes reached significance at FDR less than 0.05, with the majority showing increased expression in the older age group. We then examined the expression of these DEGs across all defined age groups (Figure 5B and Supplemental Table 9), revealing that many of the identified genes show progressive changes with age rather than abrupt shifts at a single transition point. The 2 genes with the most significant adjusted *P* values were *AKAP5* (A-kinase anchoring protein 5), which decreased with age, and *CDKN1A* (p21), which increased (Figure 5, C and D). We note that these genes, although identified as DEGs in the human dataset, did not reach significance in the mouse OVX model, which may reflect species-specific regulation, differences in statistical power between the datasets, or the distinct nature of surgical versus natural estrogen withdrawal. Spatial transcriptomic visualization of *Akap5* and *Cdkn1a* from the Allen Brain Atlas confirmed their expression in hypothalamic subregions including the dorsomedial hypothalamic nucleus, ventromedial hypothalamic nucleus, and arcuate hypothalamic nucleus (Supplemental Figure 8). Additionally, we assessed expression of canonical aging-associated genes (36) across our groups (Supplemental Figure 9A) and plotted trajectories for genes with previously reported functions in brain aging and cognitive decline (37–41) (Supplemental Figure 9B).

### Cross-species comparison reveals conserved hypothalamic inflammatory signatures.

**Figure 6:**
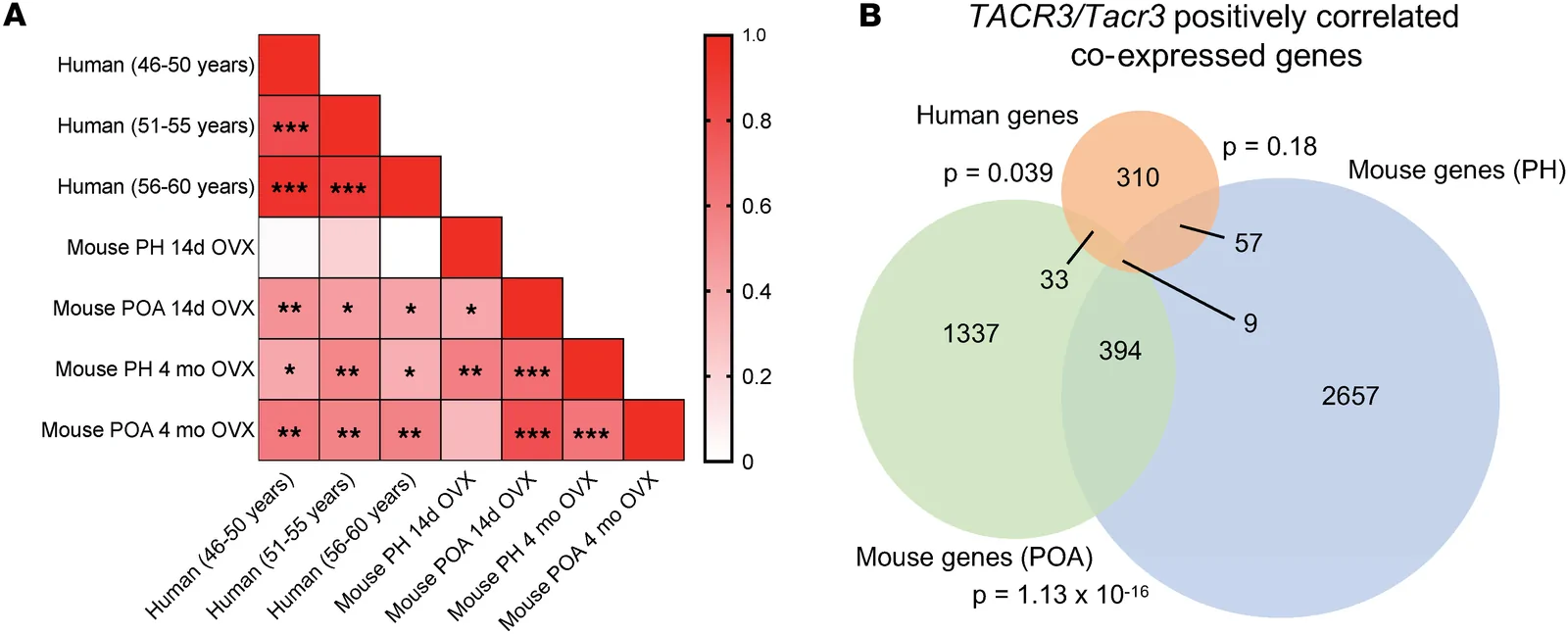
Cross-species comparison of hypothalamic transcriptomic changes. (**A**) Heatmap of GSEA pathway correlations between human age groups and mouse OVX conditions; *P* value from Pearson’s correlation (*P < 0.05; **P < 0.001; ***P < 0.0001). (**B**) Overlap of *TACR3/Tacr3* positively correlated coexpressed genes between human and mouse; *P* value from hypergeometric test.

A substantial proportion of expressed genes are shared between the human and mouse hypothalamus (~77%; Supplemental Figure 10). To assess whether the transcriptomic changes observed in the mouse OVX model capture similar changes to those identified in the human hypothalamus across age groups, we compared GSEA results between the 2 datasets. This analysis revealed statistically significant pathway enrichment correlations (Figure 6A), with the strongest cross-species correlations observed between the 4-month post-OVX time point in mice and the 51–55 age group in humans, an association most pronounced in the PH (*P* < 0.001), which was the most significant of the PH correlations across human age groups; the corresponding correlations between the 4-month mouse POA and the human 46–50, 51–55, and 56–60 age groups were comparable (all *P* < 0.001). This supports the proposition that the long-term OVX model captures core transcriptomic features that parallel age-related changes in the human female hypothalamus, though we recognize that the human changes may reflect both estrogen loss and other age-related processes.

We also compared GSEA results between our OVX mouse model and a published single-cell RNA-seq dataset of the aging female mouse hypothalamus (20) to assess whether the OVX model captures similar hypothalamic changes to those associated with natural aging in mice. Although this comparison is limited in part by methodological differences between the datasets, including bulk versus single-cell RNA-seq approaches and the aggregation of multiple cell subtypes in the single-cell dataset, of the 26 Hallmark pathways significantly affected in the human dataset (Supplemental Table 7), only 8 were similarly altered in the aged mouse hypothalamus (20) compared with 14 in the OVX model. Notably, the aged mouse study (20) did not report any changes in the estrogen response or TNF-α signaling pathways, which were consistently affected in both the human dataset and the OVX mouse model and are directly relevant to reproductive aging (42). At the cell-type level, the observed pathway changes in macrophages and microglia from the aged mouse dataset (20) exhibited the greatest similarity to those changes observed in the human and OVX mouse models (Supplemental Table 10). Despite this partial overlap, the 2 mouse models share a single significantly differentially expressed gene (*Thsd4*), suggesting that they may capture largely distinct hypothalamic transcriptional programs, the OVX model preferentially modeling estrogen-dependent changes and the aging model capturing broader senescence-related alterations.

To determine whether cross-species similarities extend beyond pathway-level analyses, we selected *TACR3*, given its documented role in VMS onset and severity (43), and performed coexpression analyses. *TACR3/Tacr3* coexpression analysis identified genes whose expression positively correlated with *TACR3* in human and *Tacr3* in the OVX mouse POA, revealing statistically significant overlap in the positively coregulated gene sets (hypergeometric test, *P* = 0.039; Figure 6B and Supplemental Table 11). We note that *TACR3/Tacr3* itself was not identified as a DEG in either species in the pairwise comparisons, but that its coexpression network is conserved, suggesting that the broader transcriptional program in which it participates is similarly affected by estrogen loss across species.

**Figure 7:**
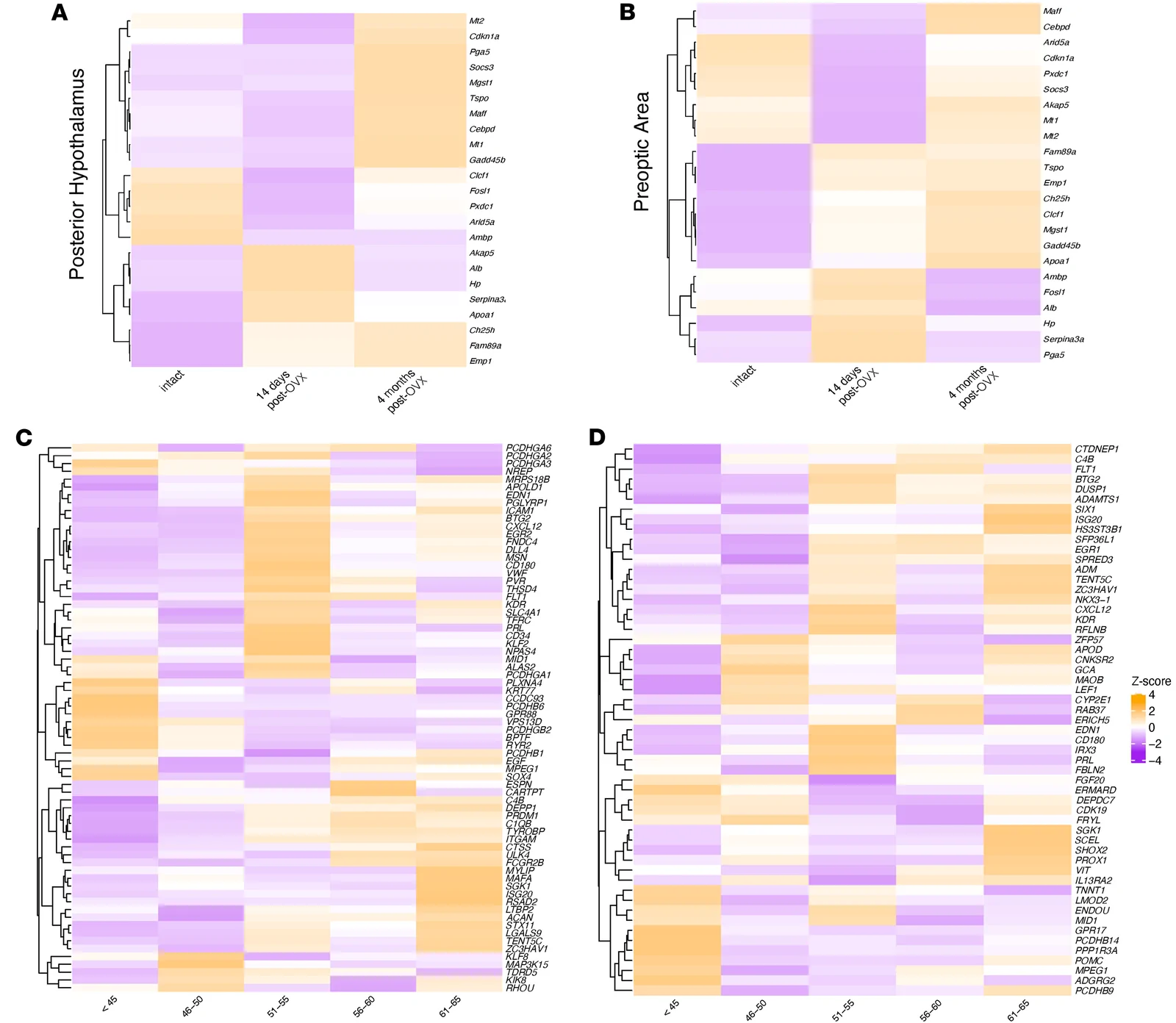
Cross-species expression of DEGs. (**A** and **B**) Expression of identified DEGs from the human dataset across conditions in the mouse (**A**) PH and (**B**) POA. (**C**) Expression of identified DEGs from the mouse PH across age groups in the human dataset. (**D**) Expression of identified DEGs from the mouse POA across age groups in the human dataset. For all panels, DEGs were identified at FDR less than 0.05 in their dataset of origin; heatmaps display *z* score expression to illustrate the concordance of direction across species and do not constitute an independent test of significance in the second species.

Finally, we examined whether DEGs identified in one species showed concordant changes in the other. When human DEGs were examined across mouse conditions, two-thirds of the genes differentially expressed in the 51–55 age group showed a clear increase at 4 months after OVX, and the remaining third were captured at 14 days after OVX, in both the PH and POA (Figure 7, A and B). The majority of mouse PH and POA DEGs that showed the highest changes at 4 months after OVX were upregulated in the 51–55 age group (Figure 7, C and D), though individual genes such as *AKAP5* and *CDKN1A* (identified as human DEGs) were not differentially expressed in the mouse, underscoring the expected species-specific differences alongside the broader pathway-level conservation.

## Discussion

This study provides a systematic temporal characterization of hypothalamic transcriptomic changes after estrogen withdrawal, revealing progressive neuroinflammation that develops over months in the mouse and whose molecular characteristics parallel age-related changes in the female human hypothalamus.

A central finding is that the hypothalamic response to estrogen loss is markedly time dependent. Although physiological changes (i.e., elevated LH and core temperature) manifest rapidly after OVX, the full transcriptomic inflammatory signature does not emerge until 4 months after OVX. This dissociation between acute neuroendocrine responses and delayed transcriptomic remodeling has important implications since short-term OVX studies, which constitute the majority of the literature, may capture the initial endocrine disruption but miss the progressive neuroinflammatory processes that could underlie the chronic sequelae of estrogen withdrawal. The delayed onset of robust inflammatory pathway activation, particularly TNF-α signaling via NF-κB and IFN responses, suggests that neuroinflammation is not a direct consequence of estrogen loss but rather develops as a secondary process, possibly driven by cumulative glial activation and loss of estrogen’s antiinflammatory protective effects.

Our protein-level validation in the arcuate nucleus provides cellular resolution to complement the transcriptomic data. The finding that NKB peptide stores are depleted while *Tac2* mRNA is increased is consistent with a state of hyperactivated neuropeptide release in KNDy neurons, particularly at 14 days after OVX. The subsequent approximately 50% decline in the KNDy (NKB^+^) activated (c-Fos^+^) arcuate cells at 4 months aligns with the normalization of core temperature at this time point and offers a potential cellular mechanism for the resolution of VMS-like thermoregulatory disruption. This temporal pattern is consistent with clinical observations that hot flashes, although beginning during perimenopause, typically resolve within 5–10 years after menopause (18), and may reflect adaptive processes within KNDy neurons to prolonged estrogen deficiency. In parallel, the progressive increase in GFAP immunoreactivity demonstrates that astrocytic reactivity continues to build even as acute neuronal hyperactivation resolves, pointing to distinct temporal dynamics for neuronal versus glial responses to estrogen withdrawal.

The concurrent increase in inflammatory signaling and astrocyte/microglia markers has functional implications beyond the hypothalamus. Activated astrocytes and microglia in thermoregulatory and metabolic hypothalamic nuclei can modulate local neuronal excitability through altered neurotransmitter reuptake, gap junction coupling, or cytokine release (44). The prostaglandin pathway changes we observed directly link to autonomic output controlling cutaneous vasodilation, the effector mechanism of VMS (45, 46). Furthermore, hypothalamic inflammation can influence systemic metabolic regulation through altered brown adipose tissue thermogenesis, potentially contributing to weight gain commonly observed after menopause (47). The upregulation of NF-κB–mediated inflammatory signaling may also propagate to peripheral tissues through neuroendocrine and autonomic pathways, contributing to the systemic low-grade inflammation (“inflammaging”) associated with cardiometabolic risk in postmenopausal women (48).

To place our experimental findings in a translational context, we analyzed human hypothalamic transcriptomic data from the GTEx consortium. Although this observational dataset has important limitations based on the modest cohort size (*n* = 31), reliance on chronological age as a proxy for menopausal status, use of bulk RNA-seq in a heterogeneous tissue, and inability to control for hormone therapy or menopausal symptoms, it represents the most comprehensive publicly available resource for studying human hypothalamic gene expression across the female life span. Thus, we explicitly framed the human data as age-related transcriptomic changes rather than menopausal changes.

Despite these caveats, the convergence between mouse and human datasets at the pathway level is notable. The inflammatory pathway enrichments observed in the 51–55 age group closely mirror those seen at 4 months after OVX, and GSEA correlation analyses confirmed significant pathway-level similarity between the long-term OVX mouse PH and the human hypothalamus during the age range typically corresponding to the late perimenopause and early postmenopause stages. That two-thirds of DEGs in the human dataset showed concordant changes at 4 months after OVX, rather than at 14 days, further supports the idea that the long-term OVX model preferentially captures the transcriptomic state associated with the later stages of reproductive aging.

The human data also revealed genes of interest, including *AKAP5* and *CDKN1A* (p21), as the most significantly differentially expressed between the 51–55 and younger than 45 age groups. *AKAP5* encodes a scaffolding protein involved in synaptic plasticity (49–52) and showed decreased expression with age in the hypothalamus. *CDKN1A*, encoding the cell-cycle regulator p21 (53), was upregulated and may promote senescence-associated secretory phenotype (53–56), potentially contributing to the inflammatory phenotype observed. Notably, these genes were not differentially expressed in the mouse OVX model, which could reflect species-specific regulation, differences in the nature of surgical versus natural estrogen withdrawal, or the contributions of aging-related processes beyond estrogen loss in the human dataset. These observations highlight the complexity of disentangling estrogen-dependent from age-dependent changes and underscore the value of using multiple complementary approaches.

Our analysis of KNDy gene expression in the human hypothalamus, although interpreted with caution given the constraints of the dataset, revealed patterns consistent with known biology. *KISS1* expression appeared to increase with age during the typical menopausal years, consistent with reports of kisspeptin neuron hypertrophy in postmenopausal women (32, 33), followed by a decline. *TACR3* expression showed a distinct trajectory, increasing before the typical age of menopause and declining thereafter, which aligns with the known time course of VMS (18). Although these KNDy genes did not reach significance as DEGs after correction for multiple testing, likely reflecting the limited sample size, their expression trajectories are biologically consistent with the established literature and with our experimental findings in the mouse model. Thus, our data offer insights into the mechanisms that could mediate the decrease in LH levels and termination of hot flashes years after menopause, likely mediated by the significant drop in hypothalamic *KISS1* and *TACR3* expression (57, 58).

Regarding the choice of animal model, although multiple rodent models exist to study the consequences of estrogen loss, we selected long-term OVX because it provides precise control over the timing of estrogen withdrawal, enabling direct temporal alignment of endocrine, thermoregulatory, and transcriptomic changes. A 4-vinylcyclohexene diepoxide model offers an alternative approach that more closely mimics gradual follicular depletion (59) but introduces confounders, including variable timing of ovarian failure (15–160 days), residual androgen production (59–61), and potential off-target effects (62, 63). Our OVX model, while representing abrupt surgical menopause, provides a robust framework for studying chronic hypoestrogenism and enables the kind of precise temporal analyses we describe here. We further note that the long-term (4-month) post-OVX group was not compared with an age-matched intact control group; however, because these animals were ovariectomized at 3–4 months of age and were therefore only 7–8 months old at tissue collection (an age at which hypothalamic transcriptomic changes attributable to normal aging are minimal relative to those seen in aged cohorts), the differences we attribute to sex steroid withdrawal are unlikely to be confounded by chronological aging over this interval.

We chose to assess gene expression changes in the POA and the PH regions of the hypothalamus based on the localization of neurons regulating temperature (POA) and neurons involved in metabolic and reproductive control (PH). This approach enabled us to observe changes in prostaglandin-related signaling associated with thermoregulation in the POA and changes in inflammatory pathways, KNDy gene expression, and glial activation in the PH.

In conclusion, this study provides a temporal framework for understanding how estrogen withdrawal progressively reshapes the hypothalamic transcriptome, moving from acute neuroendocrine disruption to chronic neuroinflammation. The convergent inflammatory signatures observed across species suggest that this neuroinflammatory program is a conserved consequence of estrogen loss, offering both mechanistic insight and a preclinical platform for testing interventions. Future studies using single-cell approaches in both species will be essential to resolve cell-type–specific contributions and establish causal relationships between these transcriptomic changes and menopausal symptoms.

## Methods

### Sex as a biological variable

All human hypothalamic samples from the GTEx database were from donors recorded as female (biological sex). This study examines transcriptomic changes across the female reproductive life span. Sex was determined by GTEx based on donor records. We use the term women to reflect the clinical population most directly affected by natural menopause, while acknowledging that individuals of diverse gender identities with intact ovarian function may also experience menopausal transition.

### Mouse studies

#### Experimental design.

To investigate the effects of sex steroid withdrawal on LH levels, core temperature, and hypothalamic transcriptome profile, intact WT female mice were bilaterally ovariectomized to generate models at progressive time points after estrogen withdrawal. Blood LH levels and core temperature were measured at baseline (intact); at 7, 14, and 21 days after OVX; and at 1, 2, and 4 months after OVX. Additional females were euthanized before OVX in diestrus (intact group), after 14 days of OVX (short-term post-OVX group), or after 4 months of OVX (long-term post-OVX group), and their PH and POA of the hypothalamus were collected for transcriptome profile analysis by bulk RNA-seq.

#### OVX.

Adult (3–4 months old) female mice were bilaterally ovariectomized under isoflurane anesthesia. Mice were treated with Buprenex SR (0.1 mg/kg, s.c.) and meloxicam (5 mg/kg, s.c.) on the day of surgery. Mice were allowed to recover for at least 1 week before the start of experiments.

#### Whole blood samples and LH ELISA.

For LH measurements, a 4 μL blood sample was collected with a pipette. Whole blood was immediately diluted in 116 μL of 0.05% PBST (Boston Bio Products, BM220) containing Tween-20 (Sigma-Aldrich, P2287), vortexed, and frozen on dry ice. Samples were stored at –80°C until analyzed using an in-house sandwich ELISA as previously described (64).

#### Core temperature.

For core temperature measurements, intact female mice were implanted (i.p.) with an IPTT-300 temperature transponder (BioMedic Data Systems, Inc.). Core temperatures from the transponder were collected with a DAS-8007 reader and DASHost 8000 software (BioMedic Data Systems, Inc.).

### IHC

Animals were anesthetized with a ketamine/xylazine/saline cocktail and transcardially perfused with saline (0.9% NaCl) followed by 4% paraformaldehyde (PFA) diluted in phosphate buffer (PB) (Boston BioProducts, Inc). Brains were removed, stored in 4% PFA overnight, and then transferred into sucrose solution (30% sucrose in 0.1 M PB, Thermo Fisher Scientific) at 4°C. After sucrose infiltration, the tissue was frozen in Tissue-Tek OCT compound (Sakura Finetek, 4583) and cut on a freezing stage microtome (Microm HM 450, Thermo Fisher Scientific) into 30 μm coronal sections. Free-floating sections were washed in tris-buffered saline (TBS; Boston BioProducts, Inc) and blocked for 1 hour at room temperature in incubation solution (TBS + 0.3% Triton X-100 [MilliporeSigma] + 2% normal goat serum). Sections were subsequently incubated for 48 hours at 4°C with primary antibodies: rabbit anti-NKB (1:1,000, Novus Biologicals, NB300-201), guinea pig anti-cFos (1:1,000, Synaptic System, 226308), and mouse anti-GFAP (1:300, Cell Signaling Technology, 3670) diluted in incubation solution. Next, sections were incubated for 1 hour at room temperature with secondary antibodies: anti-rabbit Alexa Fluor 594 (1:500, Thermo Fisher Scientific, A11012), anti-guinea pig Alexa Fluor 488 (1:500, Jackson ImmunoResearch, 706545148), and anti-mouse donkey Alexa 594 (1:500, Invitrogen, A32744). Sections were mounted onto Super-frost plus glass slides (Fisher Laboratories), air dried, and cover-slipped with VECTASHIELD mounting medium with DAPI (Vector Laboratories).

### Microscopy and image analysis

The images were captured using a fluorescence microscope (Leica DM 2500) in JPG format and processed using the open-source software ImageJ (NIH). For image quantification, representative images of the areas of interest (i.e., arcuate nucleus) were delimited using as a reference the mouse section from Allen Brain Atlas (65). Both sides of the bilateral arcuate nucleus were counted on a similar number of sections and animals, and replicate values were averaged. For NKB and c-Fos colocalization, NKB immunoreactivity was measured as immunoreactive area because the dense somatic and fiber labeling precluded reliable identification of individual NKB^+^ somata, whereas c-Fos^+^ nuclei were counted as discrete activated cell bodies; colocalization was therefore expressed as the percentage of c-Fos^+^ cells that coexpressed NKB.

### RNA extraction, library preparation, and sequencing

Mouse PH and POA regions were dissected and flash-frozen prior to extracting RNA using a Zymo Quick RNA MicroPrep kit according to the manufacturer’s instructions. RNA concentration was measured using a Qubit 4.0 fluorometer and the Qubit RNA HS assay kit (Thermo Fisher Scientific). All samples had RNA integrity numbers (RINs) greater than 7 as measured on a Bioanalyzer 2100 instrument (Agilent). RNA-seq libraries were prepared using the KAPA mRNA HyperPrep–Stranded kit (Roche) and sequenced on a NovaSeq SP (Illumina) with 50 bp paired-end reads.

### RNA-seq data processing and analysis

We performed all analyses using mouse genome build mm39. To pseudoalign reads to the transcriptome annotation and estimate expression levels, we used kallisto (66) in the tximport package (v1.24.0) (67). We imported the resulting count data into R for analysis in DESeq2 (v1.36.0) (25). To adjust for unknown, unmodeled, or latent sources of noise, we performed surrogate variable analysis (SVA) using the smartSVA package (v0.1.3) (29) and used SV1 as a covariate in the differential gene expression analysis. Pseudoalignment and DESeq2 were performed on the entire transcriptome annotation, with downstream analysis in some cases restricted to protein-coding genes as annotated in GENCODE version M30 (68). GSEA was performed on preranked ordered gene lists using the fgsea package (v1.22.0) (21).

### Prediction of protein interactions

The first 30 leading-edge genes from the comparison of the POA between female gonadal intact versus 4-month post-OVX mice were selected as the target gene list for TNF-α signaling through NF-κB. The gene list was imported into STRING (22) for network reconstruction, visualization, and enrichment analysis. Medium confidence was selected as the minimum required interaction score (0.400). Enrichment biological processes defined by gene ontology (GO) were assessed.

### Human studies

#### Human data analysis.

After subsetting the GTEx RNA expression file for our samples, we adjusted for latent sources of noise using SVA with the smartSVA package (v0.1.3) (29). Differential expression analyses across age bins were performed using DESeq2 (v1.36.0) (25), and GSEA was performed on preranked ordered gene lists using the fgsea package (v1.22.0) (21). Gene expression trajectories across age were performed on protein-coding genes using LOESS similarly to Piehl et al. (69). Gene-level output was subjected to expression trajectory correlation analysis; genes were ranked based on their correlation with *TACR3/Tacr3* expression pattern. STRING interaction analyses were performed as described above.

Given the unavailability of hormone measurements in GTEx, we used chronological age bins as proxies, informed by epidemiological data from SWAN, where median age at final menstrual period is 51–52 years (31). Samples were grouped into 5 age ranges: younger than 45 years, 46–50 years, 51–55 years, 56–60 years, and 61–65 years. We acknowledge that individual variation in menopausal timing means our age-based classification represents chronological categories, and we cannot definitively assign clinical menopausal staging or VMS status to individual donors. The GTEx consortium did not collect serum samples, precluding direct hormone measurements.

### Cell-type decomposition analysis

Cell-type decomposition analyses to estimate cell-type composition of bulk RNA-seq datasets used the BisqueRNA package (v1.0.5) (70). Supplemental Table 5 lists the marker genes used to annotate cell types (20).

### Statistics

Statistical tests are indicated in methods, figure legends, or text. All statistics were calculated using R software (v4.2.1) unless stated otherwise. Differential gene expression used DESeq2 with Benjamini-Hochberg FDR correction; genes with adjusted *P* values less than 0.05 were considered significant. SVA was performed using smartSVA. GSEA used the fgsea package on preranked gene lists, with FDR-corrected normalized enrichment scores reported. LOESS trajectory analysis was adapted from Piehl et al. (69). Statistical comparisons of physiological measurements (LH, core temperature) and immunofluorescence quantification used 1-way ANOVA with Tukey’s post hoc test or Mann-Whitney *U* test as appropriate. *P* less than 0.05 or FDR less than 0.05 was considered statistically significant.

### Study approval

#### Human hypothalamus inclusion criteria.

Bulk RNA-seq read count data from deidentified human hypothalamus tissue were obtained from publicly available GTEx project (28) data. Donor tissue was included if the following conditions were met: (a) donor eligibility criteria (INCEXC) == TRUE, (b) tissue RNA quality score (RIN) >= 6, (c) donors were female (SEX == 2).

#### Mouse studies.

The animal studies were approved by the Brigham and Women’s Hospital IACUC in the Center for Comparative Medicine. Adult WT C57BL/6 female mice were bred in-house and group-housed under constant conditions of temperature (22°C–24°C) and light (12-hour light/12-hour dark cycle), fed with standard mouse chow, and given ad libitum access to tap water.

### Data availability

Raw RNA-seq data from mouse PH and POA regions have been deposited in NCBI’s Gene Expression Omnibus (GSE288245). This paper analyzed publicly available GTEx consortium data available on the GTEx Portal (https://www.gtexportal.org/home/). Original code to perform analyses has been deposited at GitHub (https://github.com/jcbloom/hypo_menopauseNavarro). In addition to the supplemental materials, the Supporting Data Values file accompanying the figures has also been provided. Any additional information required to reanalyze the data reported in this study is available from the corresponding author upon request.

## Author contributions

JCBB, ET, SAP, and VMN contributed to conceptualization of the study. JCBB, ET, SAP, LAS, and ANF were responsible for the methodology. JCBB, ET, and SAP conducted the investigation. JCBB, ET, and SAP contributed to visualization. DCP and VMN supervised the study. JCBB and VMN wrote the original draft. JCBB, ET, SAP, HJ, DCP, and VMN reviewed and edited the manuscript.

## Conflict of interest

The authors have declared that no conflict of interest exists.

## Funding support

This work is the result of NIH funding, in whole or in part, and is subject to the NIH Public Access Policy. Through acceptance of this federal funding, the NIH has been given a right to make the work publicly available in PubMed Central.

- NIH HD090151, DK133760, and HD099084, and NIH Research Grant U54 AG062322 (ROSA Center) funded by the National Institute on Aging and Office of Research on Women’s Health (to ET, SAP, LAS, ANF, and VMN).
- Brit Jepson d’Arbeloff Center on Women’s Health (to JCBB and DCP).

## Supplementary Material

Supplemental data

Supplemental table 1

Supplemental table 2

Supplemental table 3

Supplemental table 4

Supplemental table 5

Supplemental table 6

Supplemental table 7

Supplemental table 8

Supplemental table 9

Supplemental table 10

Supplemental table 11

Supporting data values

## Version 1 07/23/2026

In-Press Preview

## Version 2 09/08/2026

Electronic publication
