# Supplementary material for: Progressive hypothalamic neuroinflammation in ovariectomized mice parallels aging-related transcriptomic changes in the female human hypothalamus
Source: JCI Insight. 2026 Jul 23;11(17):e207270. doi: 10.1172/jci.insight.207270 (PMC13564084; doi:10.1172/jci.insight.207270)
Supplement: Supplementary file 1 [file jciinsight-11-207270-s227.pdf]

Supplementary Material:

Supplementary Figure 1. Cell type deconvolution of the mouse PH and POA RNA-seq dataset using BisqueRNA with established marker genes from Hajdarovic et al. 2022 (20).

Supplementary Figure 2. STRING analysis of TNF-alpha hallmark pathway leading edge genes in the POA between a) 4-months post-OVX vs. intact and b) 14-days post-OVX vs. intact models.

Supplementary Figure 3. Volcano plots highlighting significantly differentially expressed genes between labeled conditions and regions. Genes with FDR < 0.05 are labeled in pink. a) PH 4 months post-OVX vs. 14 days post-OVX (35 protein-coding DEGs), b) POA 4 months post-OVX vs. 14 days post-OVX (15 protein-coding DEGs).

Supplementary Figure 4. Candidate gene analysis in the post-OVX mouse hypothalamus. a) Expression of inflammation-related, astrocyte and microglia marker genes in the PH (top row) and POA (bottom row). b) Expression of KNDy genes and their receptors in the PH (top row) and POA (bottom row).

Supplementary Figure 5. Overview of the GTEx donor subset used in this study. a) Demographic overview of the GTEx donor subset used in this study (n=31). b) Distribution of ages within the donor cohort and the defined age groupings for subsequent analyses.

Supplementary Figure 6. Cell type deconvolution of the human RNA-seq dataset using BisqueRNA. a) Cell type composition using established marker genes from Hajdarovic et al. 2022(20). b) Presence of cell types expressing key transcription factors from the human hypothalamus reference atlas (Tadross et al. 2025)(30).

Supplementary Figure 7. LOESS expression trajectories of additional postmortem tissue biomarker genes (*PGR*, *GPER1*, *ESR2*) examined in Tickerhoof et al. 2026 (35).

Supplementary Figure 8. Spatial transcriptomic visualization of *Akap5* (a) and *Cdkn1a* (b) from the Allen Brain Atlas Brain Knowledge Platform. Expression localization shown with darker coloring indicating regions with higher expression.

Supplementary Figure 9. Expression of previously reported aging-associated genes from the literature in the human female hypothalamus. a) Heatmap of aging-associated genes and their expression across age groups. b) Expression of reported brain-associated aging genes across age.

Supplementary Figure 10. Overlap of expressed genes between human and mouse hypothalamus tissue; p-value from hypergeometric test.

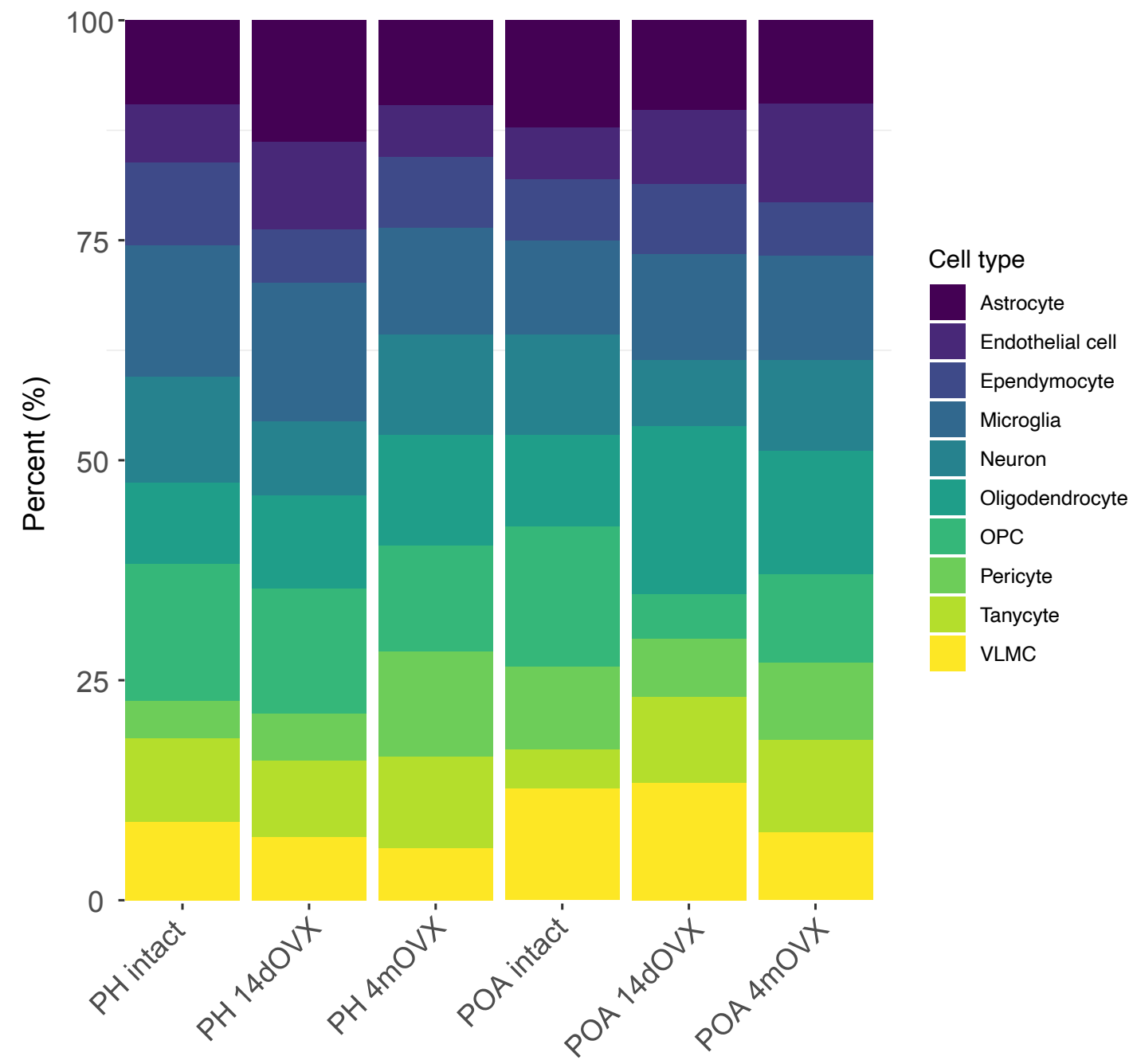

Supplementary Figure 1

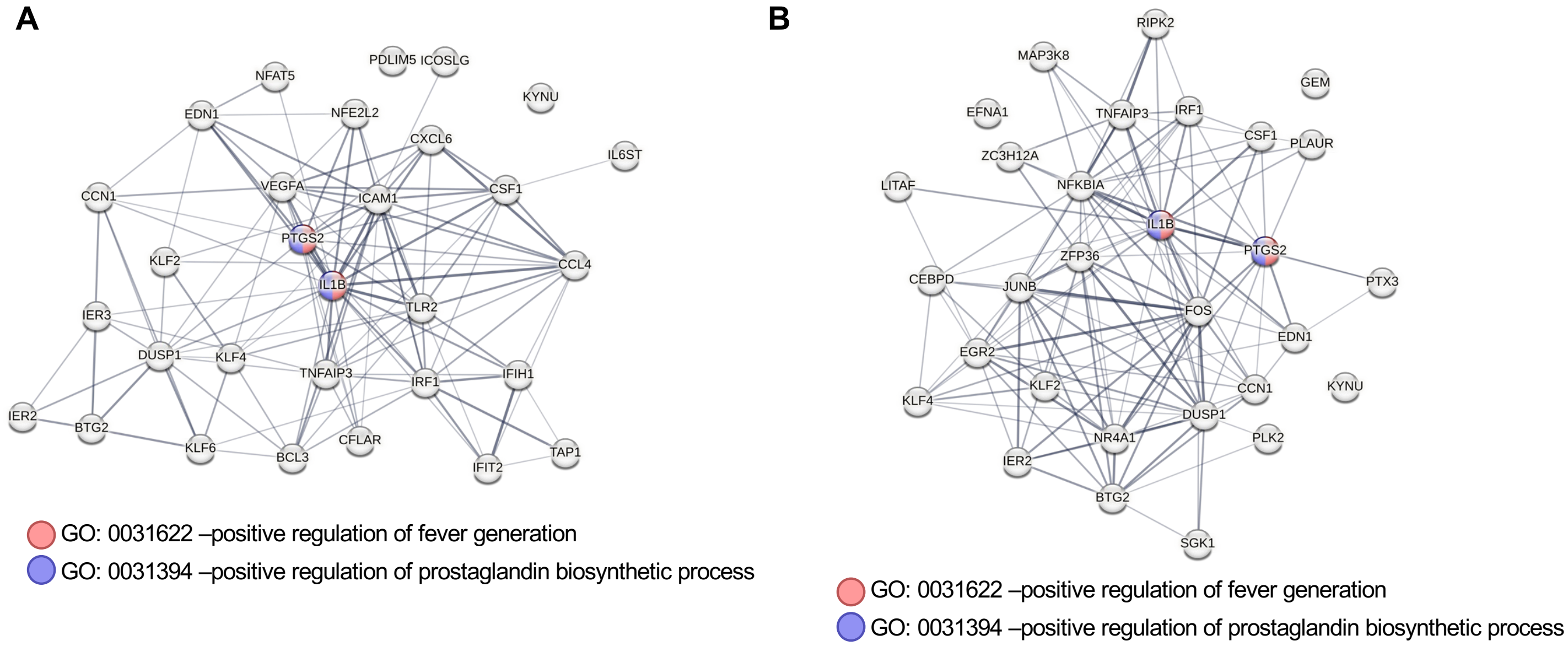

Supplementary Figure 2

A

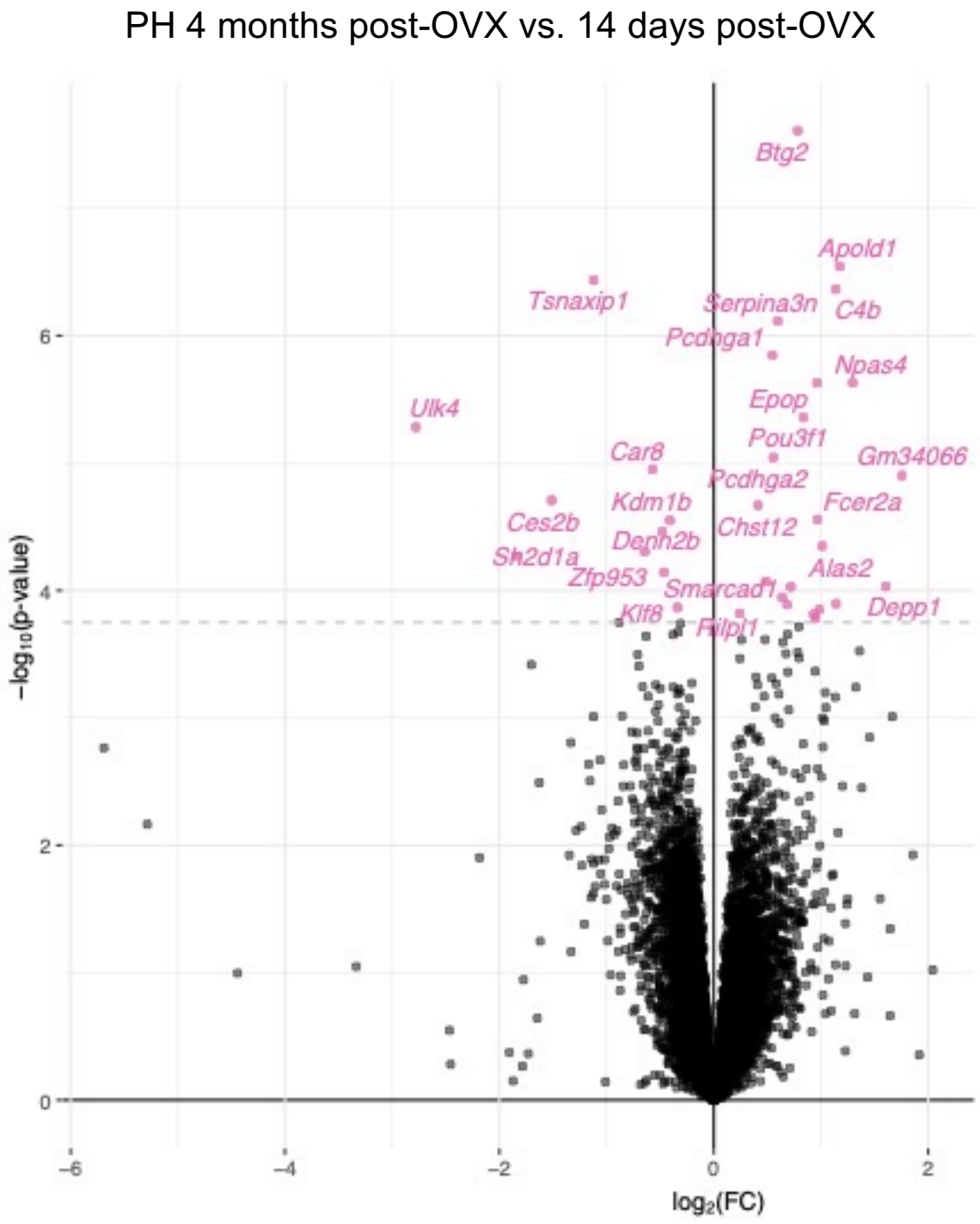

B

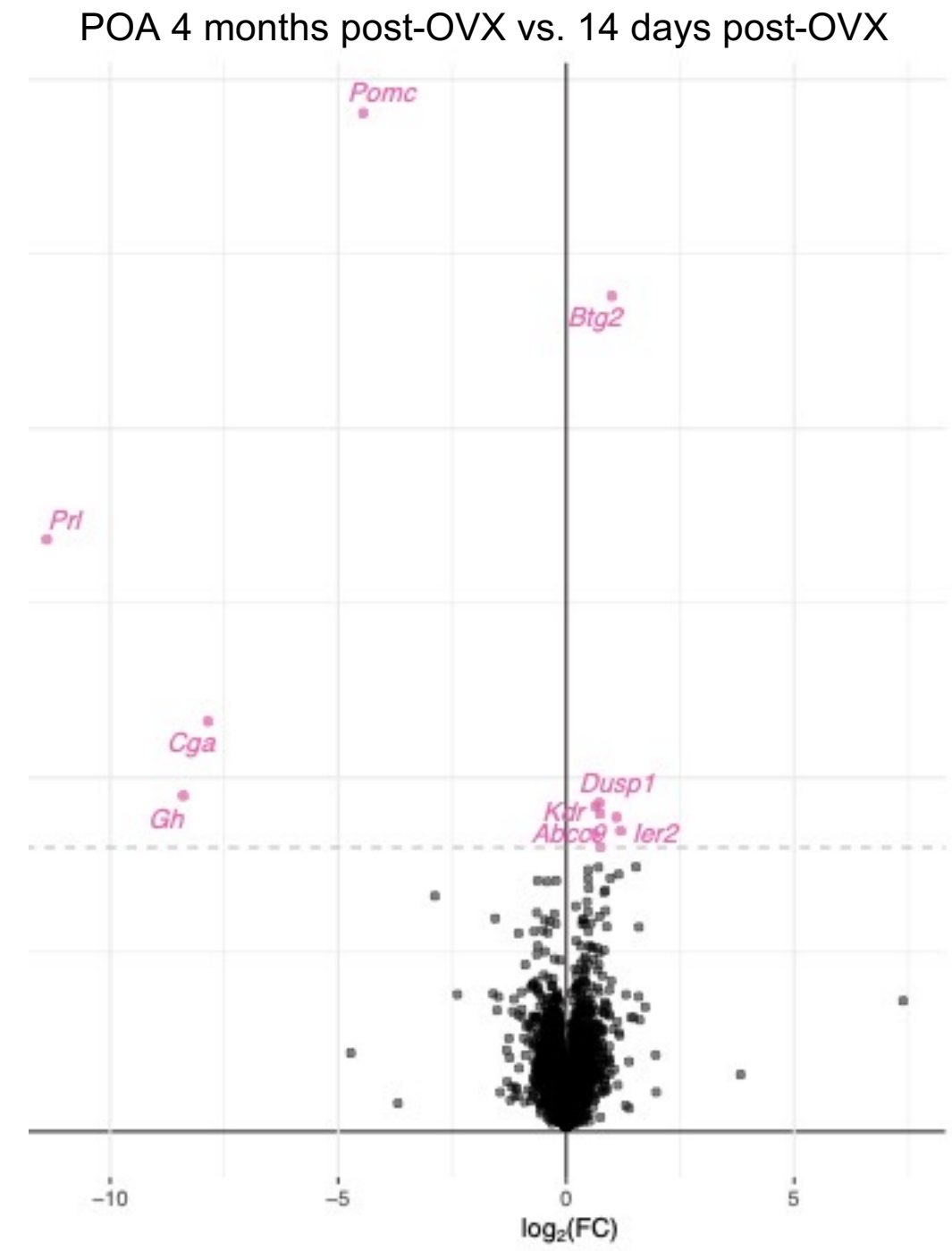

Supplementary Figure 3

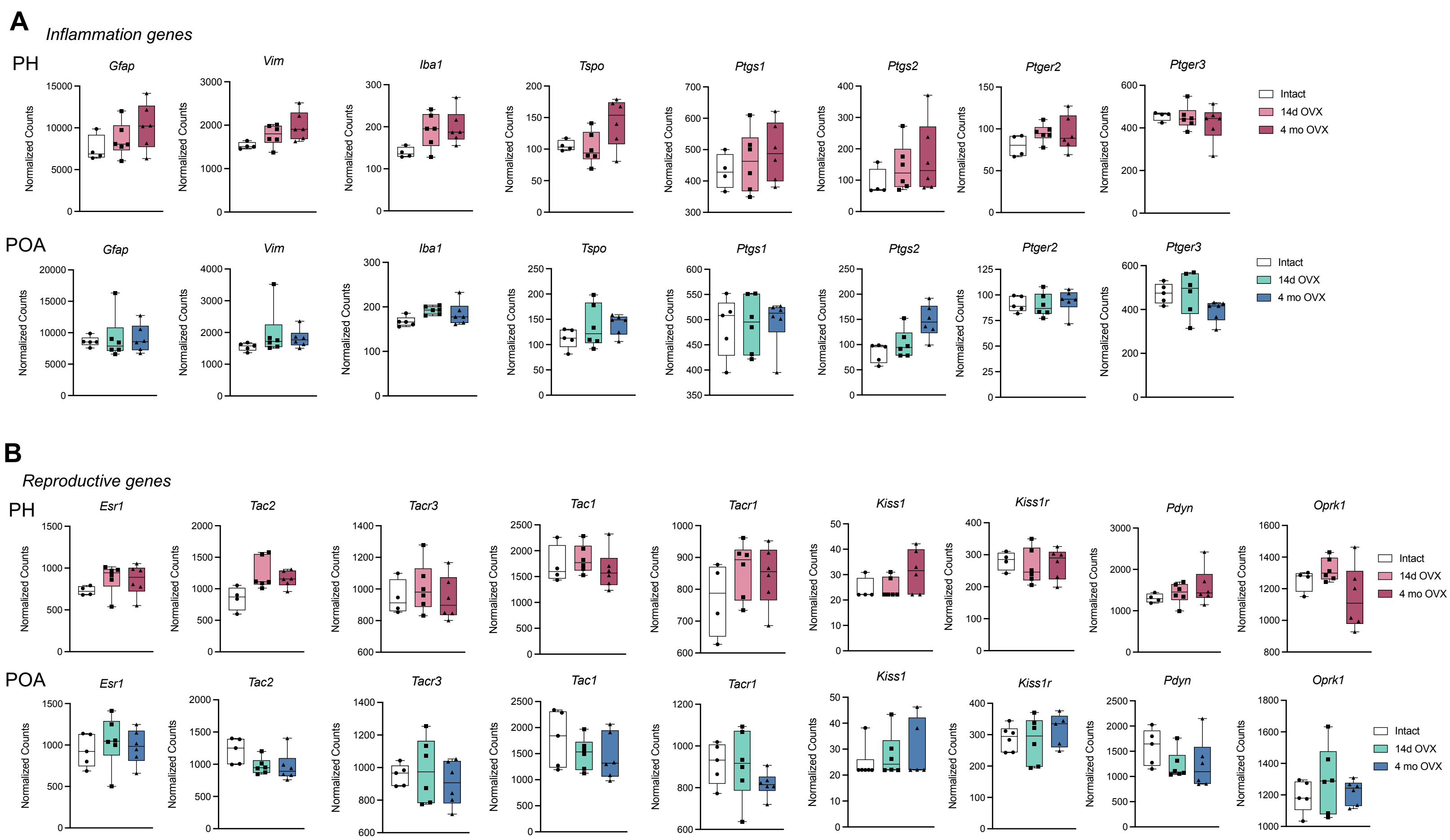

Supplementary Figure 4

A

Demographics of study subjects		
# of donor tissue		31
Age (years)	mean (min., max.)	53.7 (28, 65)
Body mass index (BMI)	mean (min., max.)	28.6 (20.1, 33,4)
Race, n (%)	Asian	1 (3.2%)
	Black/African American	2 (6.5%)
	White	28 (90.3%)

B

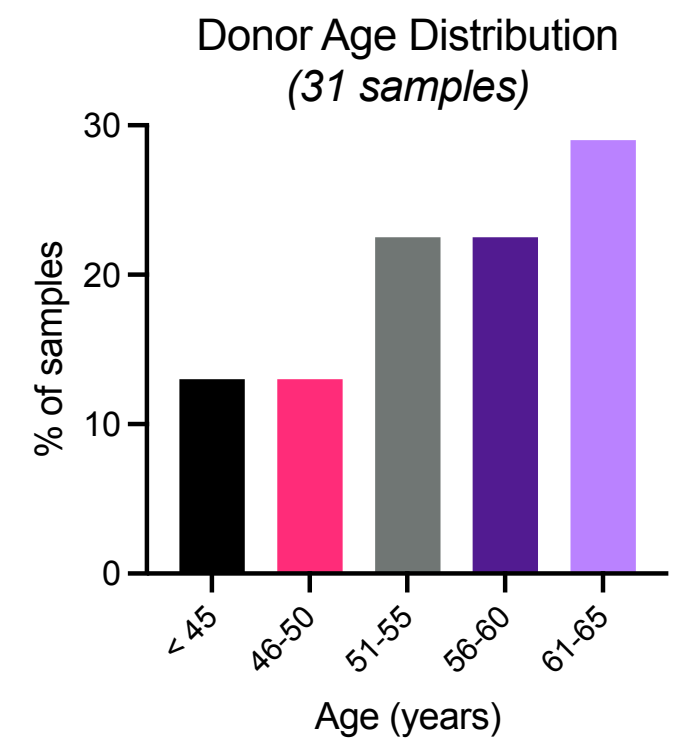

Supplementary Figure 5

A

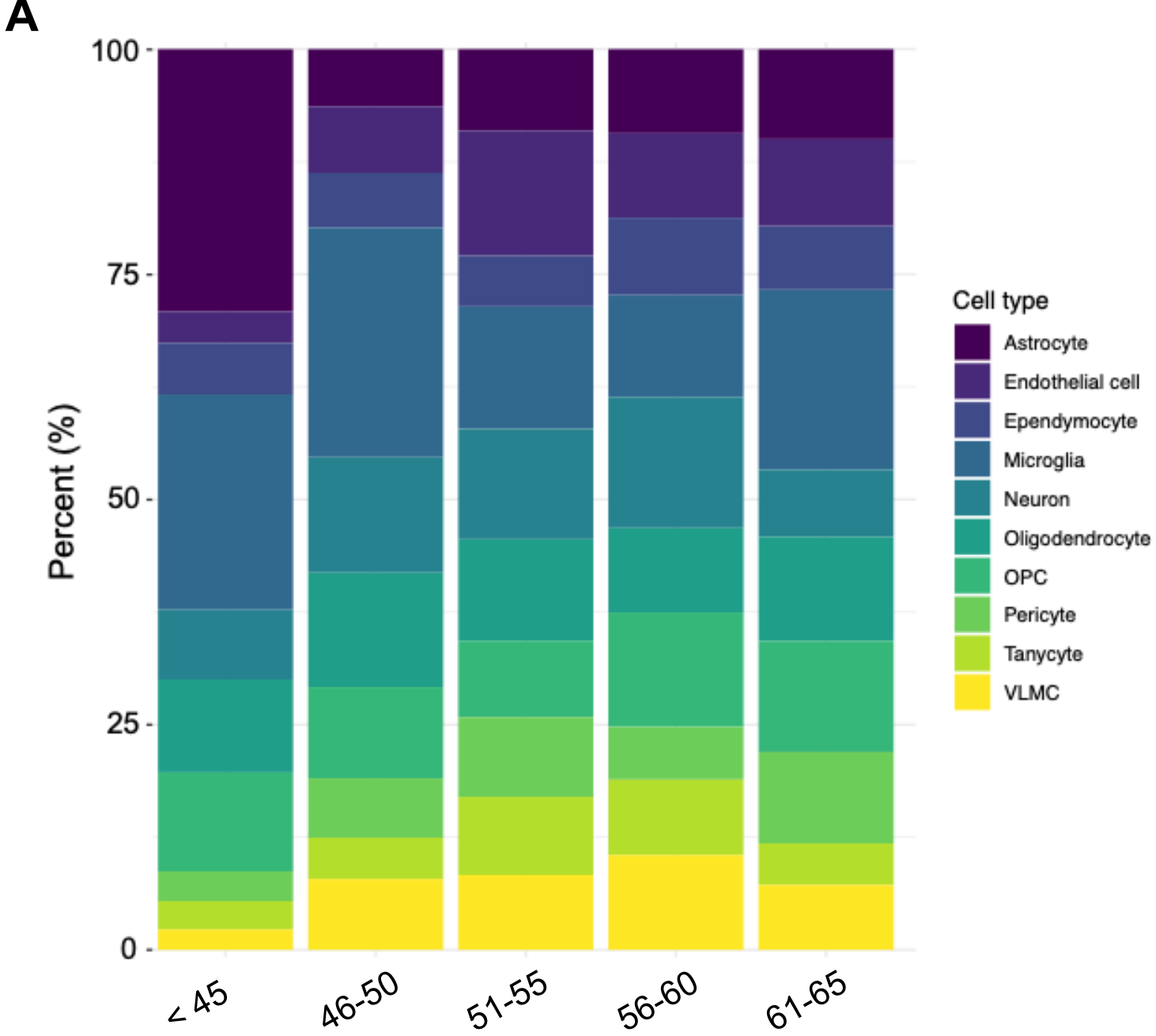

B

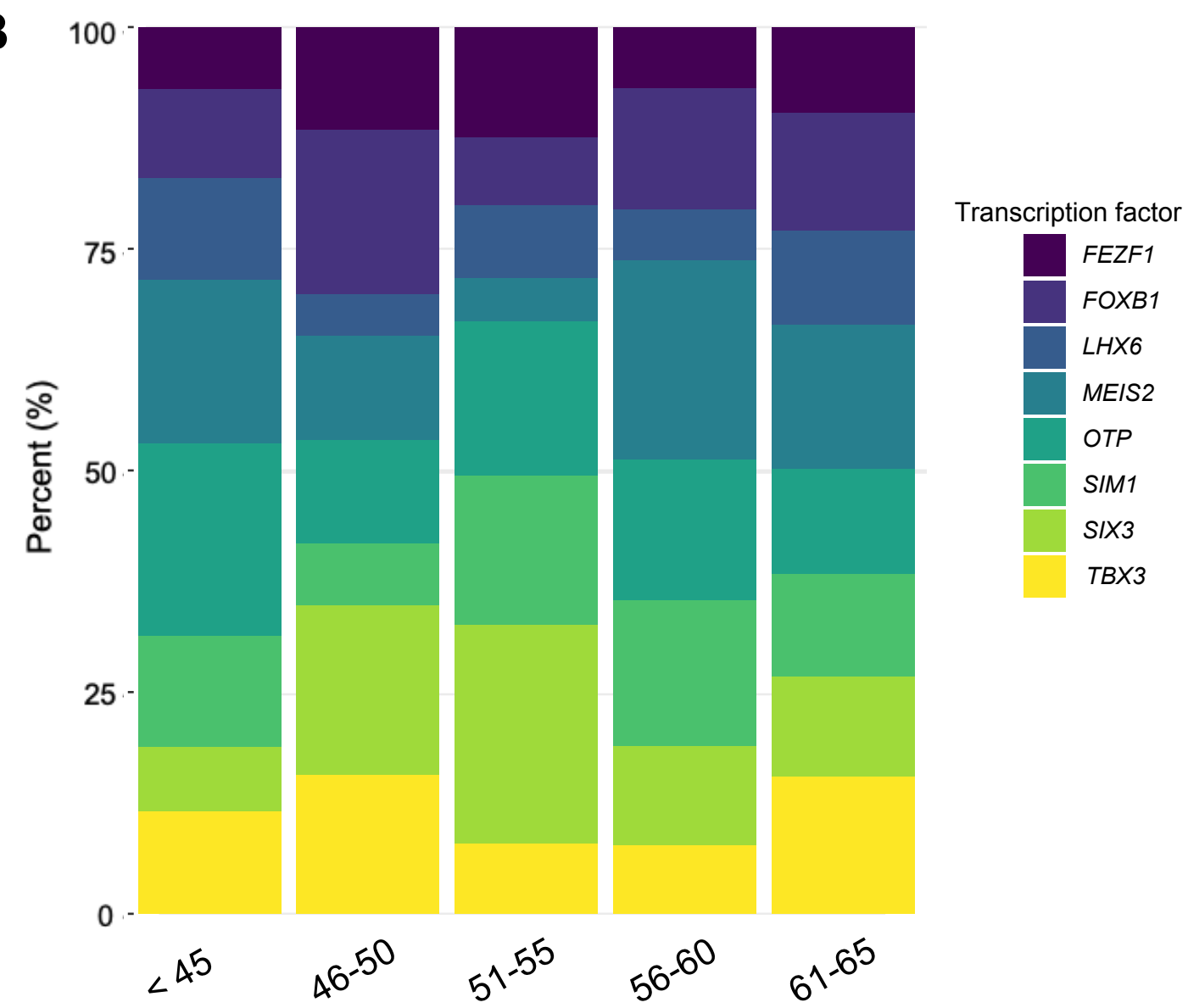

Supplementary Figure 6

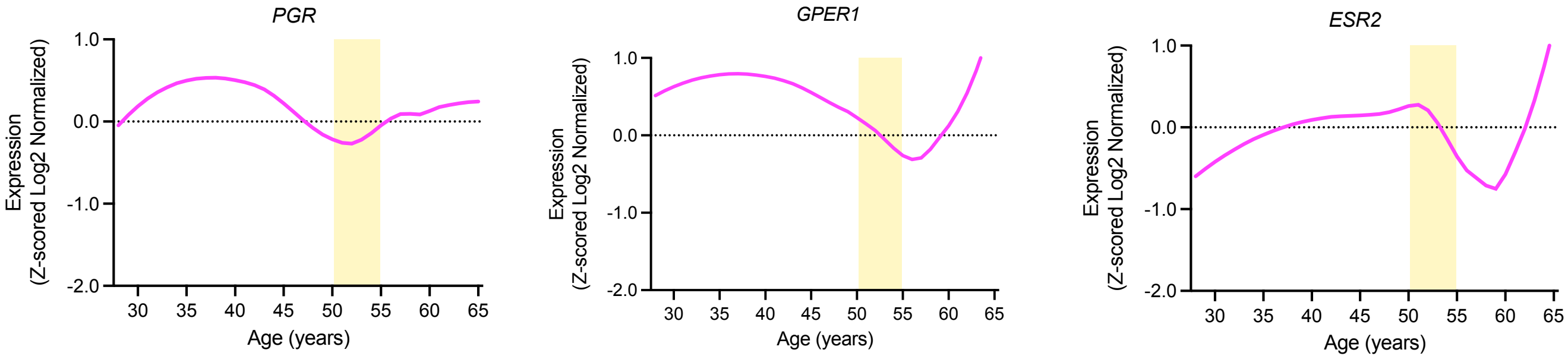

Supplementary Figure 7

A

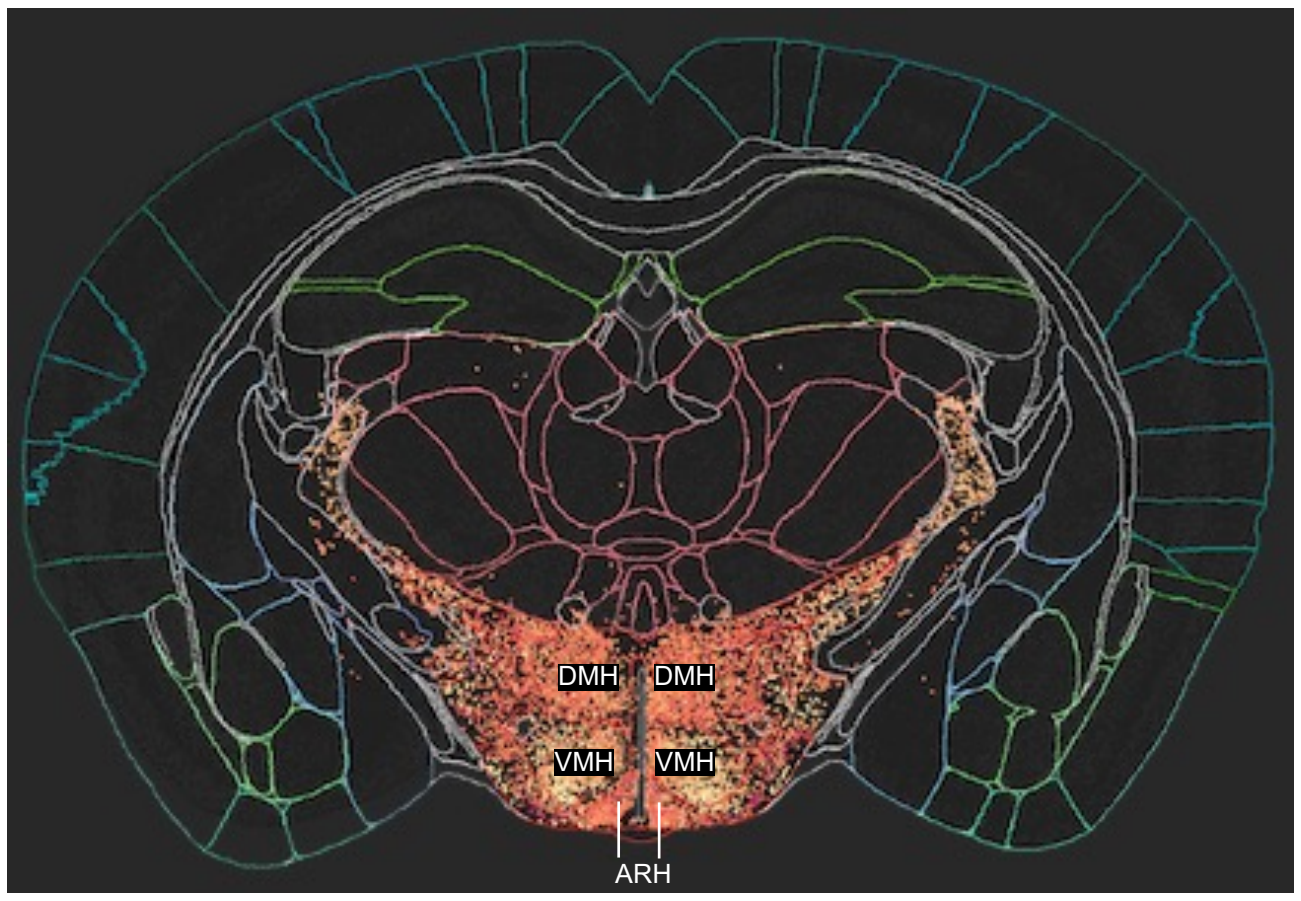

B

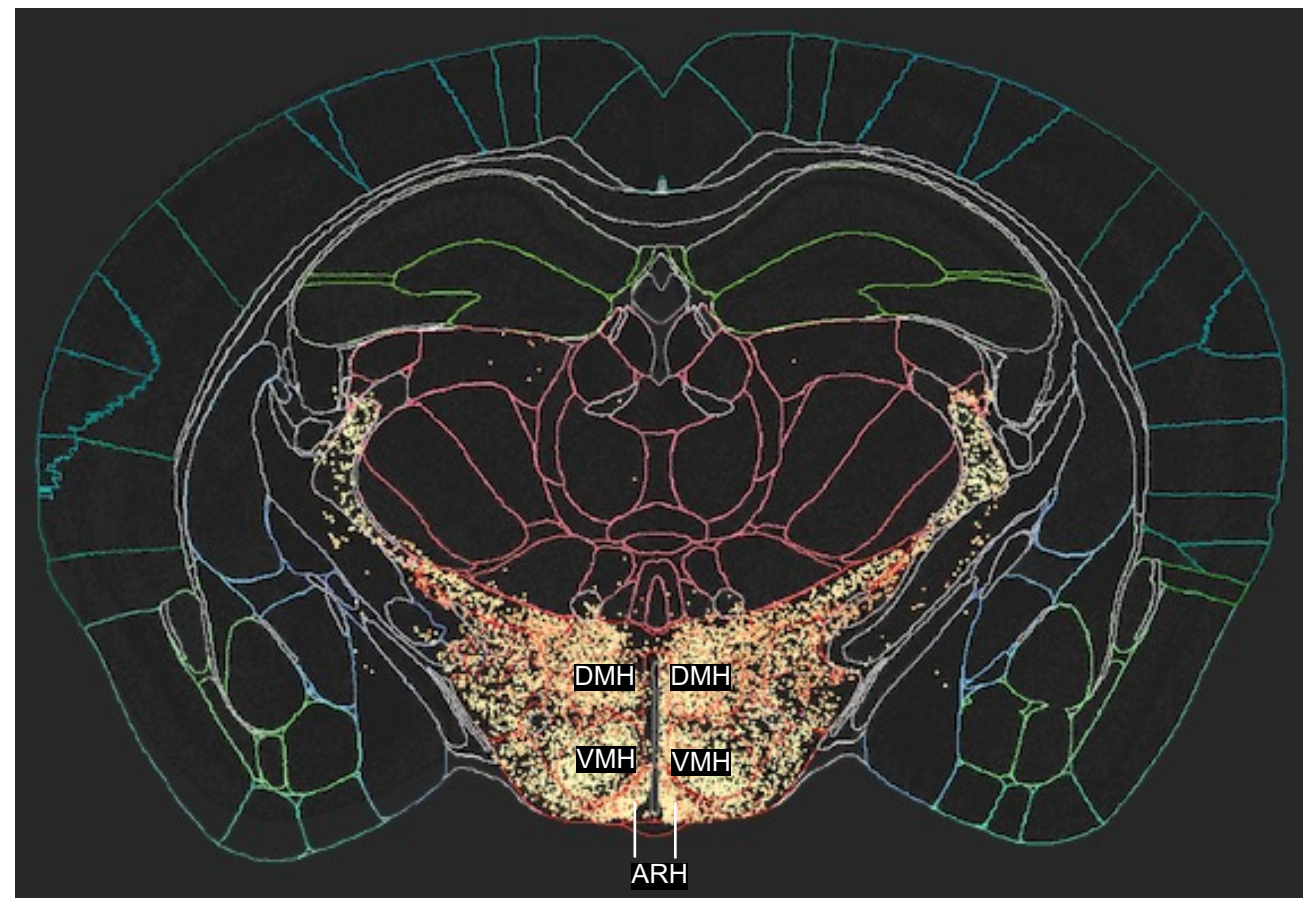

Supplementary Figure 8

A

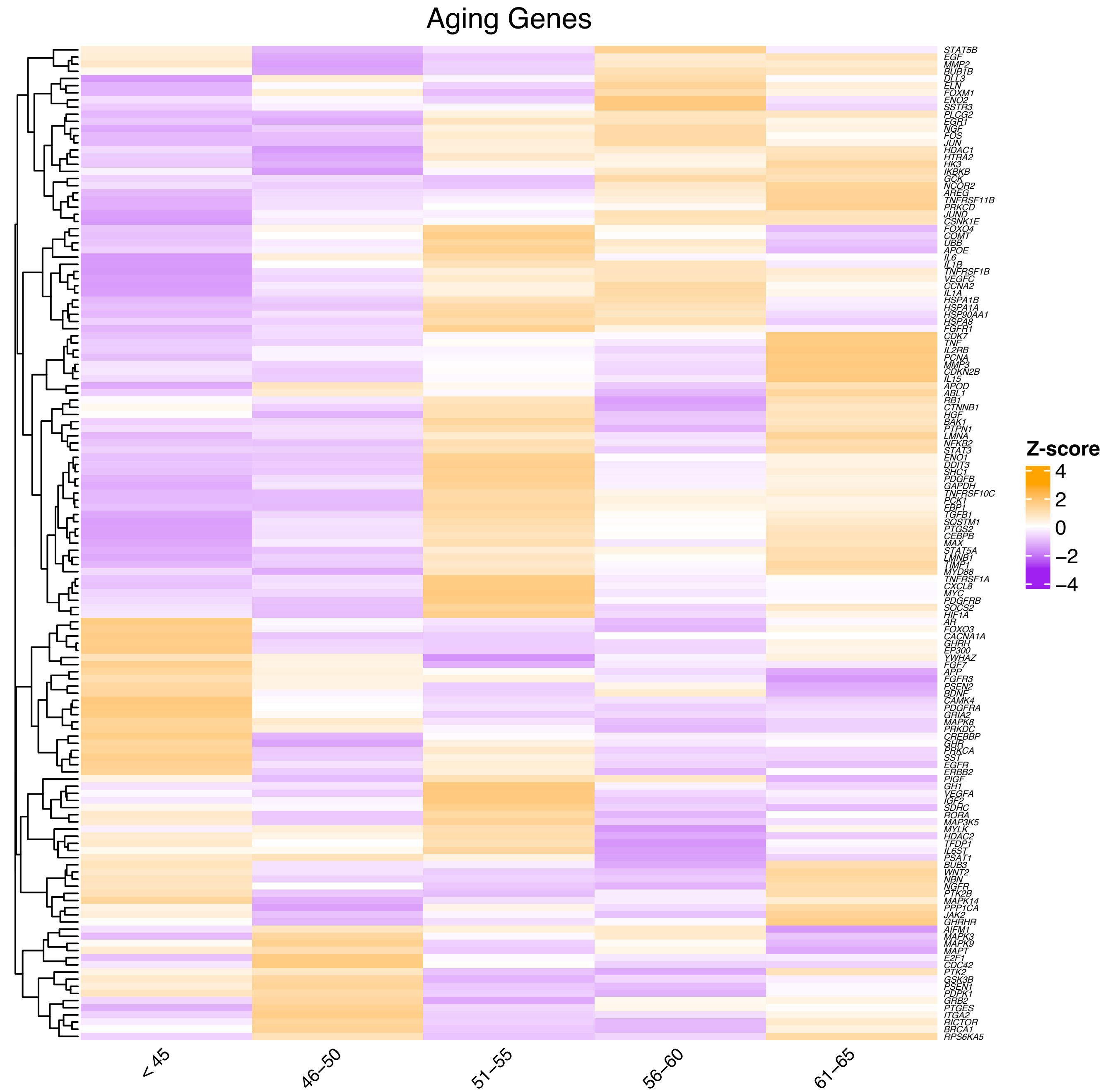

B

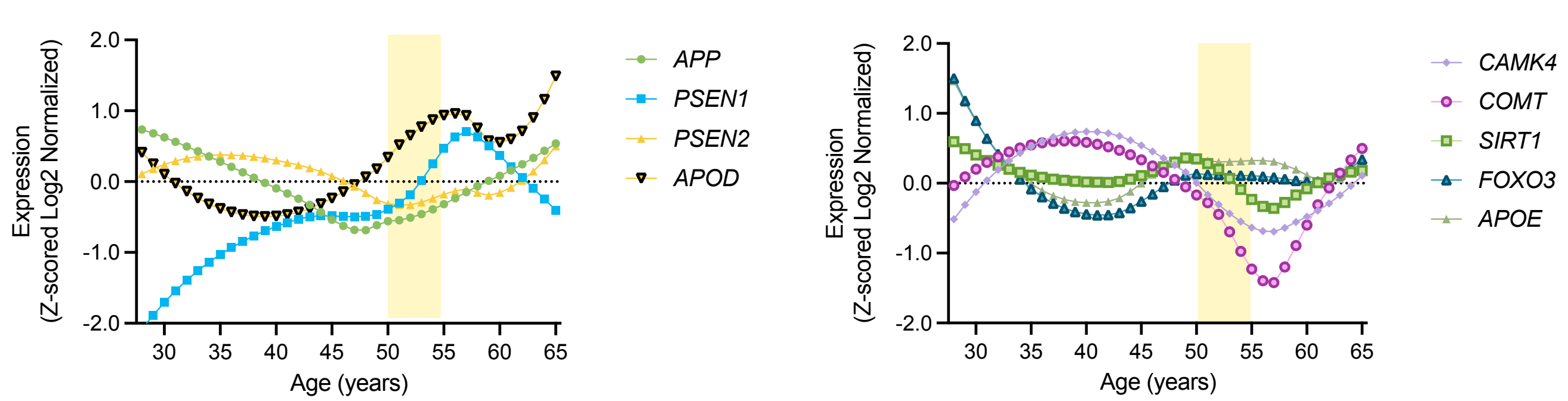

Supplementary Figure 9

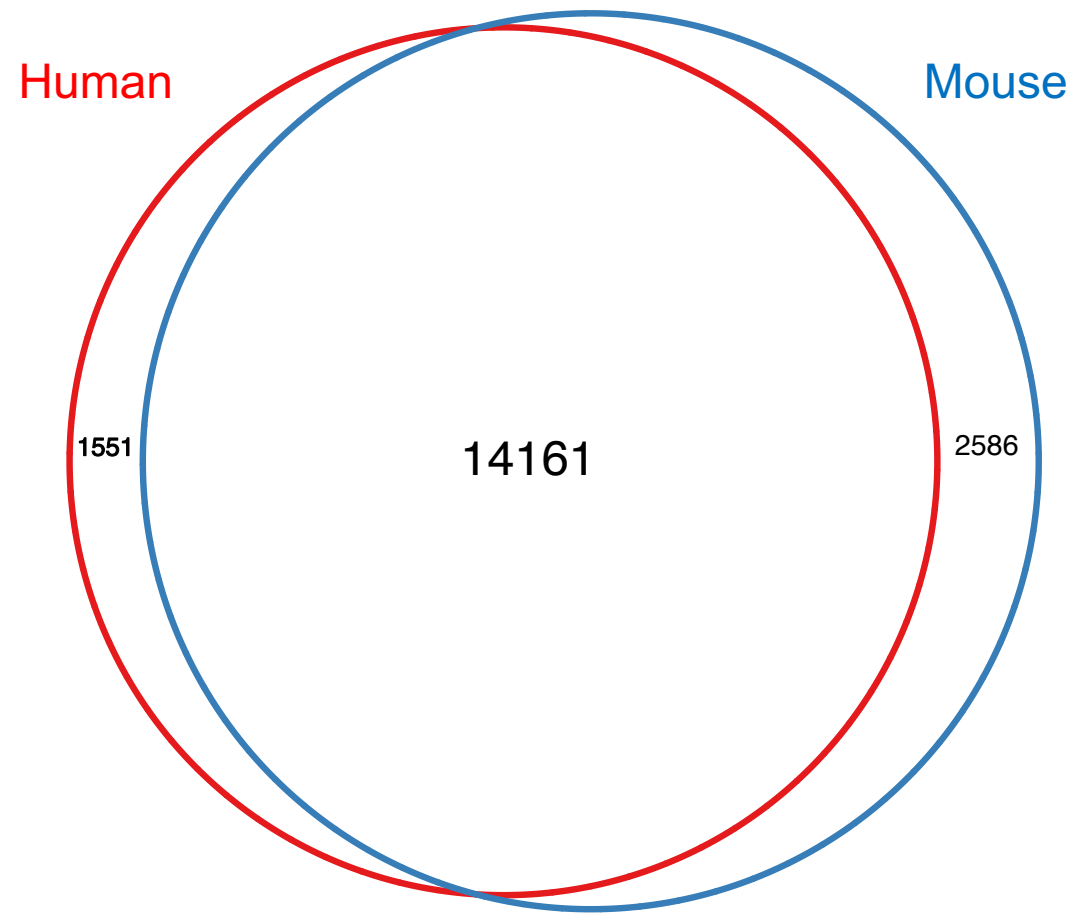

Supplementary Figure 10

Supplementary Table 1. Gene set enrichment results (mouse).

Supplementary Table 2. Differential gene expression output from DESeq2 (mouse).

Supplementary Table 3. Normalized gene expression counts (mouse).

Supplementary Table 4. GTEx donor IDs used in this study.

Supplementary Table 5. Marker genes used to annotate cell types (human and mouse).

Supplementary Table 6. LOESS regression values across age per gene.

Supplementary Table 7. Gene set enrichment results (human).

Supplementary Table 8. Differential gene expression output from DESeq2 (human).

Supplementary Table 9. Normalized gene expression counts (human).

Supplementary Table 10. Comparison of GSEA results between human, OVX mouse models (this study), and aged mice (Hajdarovic et al. 2022).

Supplementary Table 11. *TACR3/Tacr3* correlated expression ranked gene list.
